# Selective degradation of PU.1 during autophagy represses the differentiation and antitumour activity of T_H_9 cells

**DOI:** 10.1038/s41467-017-00468-w

**Published:** 2017-09-15

**Authors:** Thaiz Rivera Vargas, Zhijian Cai, Yingying Shen, Magalie Dosset, Isis Benoit-Lizon, Tiffany Martin, Aurélie Roussey, Richard A. Flavell, François Ghiringhelli, Lionel Apetoh

**Affiliations:** 1 INSERM, U1231, Dijon, 21000 France; 2 Université de Bourgogne Franche Comté, Dijon, 21000 France; 3 Institute of Immunology, Zhejiang University School of Medicine, Hangzhou, People’s Republic of China; 4 Etablissement Français du Sang, Besançon, 25000 France; 5 Department of Immunobiology, Yale University School of Medicine, New Haven, Connecticut 06520 USA; 6 Howard Hughes Medical Institute, Yale University School of Medicine, New Haven, Connecticut 06520 USA; 7 Centre Georges François Leclerc, Dijon, 21000 France

## Abstract

Autophagy, a catabolic mechanism that involves degradation of cellular components, is essential for cell homeostasis. Although autophagy favours the lineage stability of regulatory T cells, the contribution of autophagy to the differentiation of effector CD4 T cells remains unclear. Here we show that autophagy selectively represses T helper 9 (T_H_9) cell differentiation. CD4 T cells lacking *Atg3* or *Atg5* have increased interleukin-9 (IL-9) expression upon differentiation into T_H_9 cells relative to *Atg3-* or *Atg5*-expressing control cells. In addition, the T_H_9 cell transcription factor, PU.1, undergoes K63 ubiquitination and degradation through p62-dependent selective autophagy. Finally, the blockade of autophagy enhances T_H_9 cell anticancer functions in vivo, and mice with T cell-specific deletion of *Atg5* have reduced tumour outgrowth in an IL-9-dependent manner. Overall, our findings reveal an unexpected function of autophagy in the modulation of T_H_9 cell differentiation and antitumour activity, and prompt potential autophagy-dependent modulations of T_H_9 activity for cancer immunotherapy.

## Abstract (web summary)

Autophagy is a cellular process for recycling cell constituents, and is essential for T cell activation, but its function in T cell polarization is still unclear. Here the authors show that autophagy induces the degradation of transcription factor PU.1 to negatively modulate T_H_9 homeostasis and antitumour immunity.

## Introduction

Macroautophagy (henceforth referred to as autophagy) is a highly conserved catabolic mechanism responsible for organelles turnover, membrane recycling and protein degradation^1, 2^. This dynamic process involves the formation of double-membrane vesicles termed autophagosomes, which engulf portions of the cytoplasm and then fuse with lysosomes for degradation. Under cell stress, autophagy can serve as a survival mechanism allowing the reuse of cytosolic constituents^3^. Autophagy is initiated through a well-conserved molecular pathway that involves the Atg5–Atg12 covalent protein complex and microtubule-associated protein 1 light chain 3 (LC3, homologous to yeast Atg8)^4^. Subsequently, Atg16L1 promotes the conjugation of LC3 to phosphatidylethanolamine, resulting in autophagosome formation^5^.

Autophagy can also selectively target specific long-lived proteins, protein complexes, protein aggregates, various organelles and even intracellular microbes for destruction. This process, referred to as selective autophagy, can drive the degradation of specific proteins through recognition of ubiquitinated cargos via ubiquitin-binding domains of adaptor proteins^6^. Selective autophagy employs dedicated cargo adaptors such as p62, an adaptor protein that specifically targets, recruits and degrades a certain type of cargo in response to various intracellular or extracellular signals^7^. These adaptor proteins bind simultaneously to cargo and lipidated LC3 to selectively recruit the autophagy machinery to induce autophagosome formation^6, 7^. Selective degradation of key regulatory proteins in a timely and spatially regulated manner is essential for processes like gene expression, cell cycle progression, apoptosis and cell differentiation.

Autophagy has contrasting roles in cancer^8, 9^. While autophagy is required for immunogenic cell death process and acts as a tumour suppression mechanism ensuring the suppression of DNA damage and genomic instability as well as promoting protein quality control, autophagy also enables tumour cell survival in the tumour microenvironment and thereby supports tumour outgrowth^8, 9^. Accordingly, thyroid carcinoma patients carrying the autophagy-preventing single-nucleotide polymorphism (SNP) Thr300Ala of the *ATG16L1* gene have improved clinical outcomes^10^. Likewise, the *ATG16L1* Thr300Ala SNP is associated with a reduced risk of metastasis in colorectal cancer patients^11^.

Autophagy controls T-cell homeostasis, as illustrated by its requirement for the activation, proliferation and survival of T cells^12–14^. Autophagy also contributes to the formation of CD8^+^ memory T cells^15^, and couples environmental signals and metabolic homeostasis to protect lineage integrity and survival of regulatory T cells^16^. In line with this, autophagy induction following T-cell receptor (TCR) activation is shown to promote CD4 T-cell survival and antigen-dependent proliferation^17^. Autophagy is also observed in T helper 1 (T_H_1) and T helper 2 (T_H_2) cells upon differentiation from naive T cells^17^. Despite these observations demonstrating the potential relevance of the autophagic process in T cells, the functions of autophagy in effector T-cell differentiation remains incompletely understood.

Here we investigate the function of autophagy in the differentiation of effector CD4 T cells. We find that autophagy selectively restrains T_H_9 cell polarization. CD4 T cells lacking autophagy-essential genes *Map1lc3b* (encoding LC3 B), *Atg3* or *Atg5* have increased interleukin-9 (IL-9) secretion upon differentiation into T_H_9 cells. Under T_H_9-skewing conditions, the T_H_9 cell transcription factor PU.1 undergoes ubiquitination and degradation through p62-dependent selective autophagy. Importantly, inhibition of autophagy in T_H_9 cells enhances their anticancer functions in vivo, and mice with T cell-specific deletion of *Atg5* have reduced tumour outgrowth in an IL-9-dependent manner. These results implicate selective autophagy in the modulation of T_H_9 cell differentiation and prompt a potential mechanism for enhancing T_H_9 cell antitumour properties.

## Results

### Autophagy controls IL-9 secretion from T_H_9 cells

**Fig. 1:**
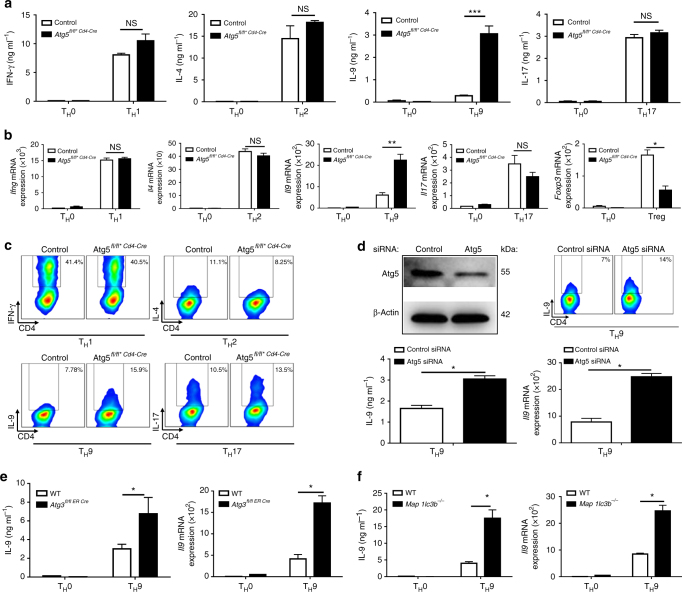
The autophagy-related proteins Atg5, Atg3 and LC3-II negatively regulate T_H_9 cell differentiation in vitro. Cell-sorted naive CD4^+^CD62L^hi^CD44^lo^ T cells were isolated from control and conditional Atg5-deficient (Atg5^fl/fl*CD4-Cre^) mice and differentiated into T_H_0, T_H_1, T_H_2, T_H_9 and T_H_17 cells in the presence of plate-bound anti-CD3 and anti-CD28 antibodies for 72 h. **a** ELISA of IFN-γ, IL-4, IL-9 and IL-17 in supernatants of T_H_0, T_H_1, T_H_2, T_H_9 and T_H_17 cells differentiated for 3 days from Atg5^fl/+*CD4-Cre^ and Atg5^fl/fl*CD4-Cre^ naive CD4^+^ T cells. **b** Quantitative RT-PCR analysis of *Ifng*, *Il4*, *Il9*, *Il17* and *Foxp3* mRNA in T_H_0, T_H_1, T_H_2, T_H_9, T_H_17 and Treg cells differentiated for 48 h from Atg5^fl/+*CD4-Cre^ and Atg5-Atg5^fl/fl*CD4-Cre^ naive CD4^+^ T cells; results were normalized to the expression of *Actb* (encoding β-actin) and are presented relative to control T_H_0 cells. Experiment performed three times. **c** Flow cytometry analysis of IFN-γ, IL-4, IL-9, and IL-17 expression from T_H_1, T_H_2, T_H_9 and T_H_17 cells differentiated for 3 days from Atg5^fl/+*CD4-Cre^ and Atg5^fl/fl*CD4-Cre^ naive CD4^+^ T cells. **d** T_H_9 cells transfected with control siRNA or Atg5 siRNA. Atg5 inhibition was assessed by western blot. IL-9 expression was analysed by ELISA, qRT-PCR and flow cytometry. Experiment performed twice. Mean (+s.d.), paired Student’s *t*-test two-tailed. **e**, **f** IL-9 expression assessed by ELISA and qRT-PCR from T_H_9 cells differentiated for 3 days from naive WT, *Atg3*-deficient (*Atg3* ^−/−^) and *Map1lc3b-*deficient (*Map1lc3b* ^−/−^) CD4^+^ T cells. qRT-PCR results were normalized to the expression of *Actb* and are presented relative to control T_H_0 cells. Data are representative of three experiments. Mean (+s.d.), NS, not significant with *P*>0.05; *P<0.05; **P<0.01; ***P<0.01; two-way analysis of variance (ANOVA) test

To test the contribution of autophagy in CD4 T-cell differentiation, we selectively suppressed T-cell expression of Atg5, a protein required for autophagy, by crossing *Atg5-*floxed mice to CD4 Cre mice and differentiated naive *Atg5*-deficient CD4 T cells (Atg5fl/fl*CD4-Cre) into T_H_1, T_H_2, T_H_17 and T_H_9 effector subsets as well as into regulatory T (Treg) cells using plate-bound anti-CD3 and anti-CD28 antibody stimulation and selective choice of differentiating cytokines (Supplementary Fig. 1a, b, c). We noted that *Atg5* deficiency failed to affect the secretion of the T_H_1 and T_H_17 cytokines interferon-γ (IFN-γ) and IL-17 respectively and modestly induced IL-4, IL-5 and IL-13 from T_H_2 cells without reaching a significant increase by enzyme-linked immunosorbent assay (ELISA) analysis of IL-4 and IL-5 cytokines (Fig. 1 and Supplementary Fig.1d). By contrast, we found that in the absence of *Atg5*, T_H_9 cell differentiation was markedly increased, as illustrated by a fivefold increase in IL-9 secretion from differentiating T_H_9 cells measured by ELISA (Fig. 1a). In line with Wei et al.^16^, we found that Treg cell differentiation was significantly decreased in the absence of *Atg5* (Fig. 1b). These results were confirmed by real-time PCR (RT-PCR) analysis and intracellular cytokine staining (Fig. 1b, c). Similar results were also obtained using CD4 T cells from *Atg5-*floxed mice (Atg5fl/fl) transduced with a Cre overexpressing vector (Supplementary Fig. 1e, f). Because it was previously described that *Atg5* affects T-cell proliferation and survival^12^, we investigated the impact of *Atg5* deficiency on T_H_9 cell proliferation and survival. We found that T_H_9 cells from Atg5^fl/fl*CD4-Cre^ mice expressed higher levels of Ki67 than those in WT T_H_9 cells, suggesting that Atg5 is dispensable for T_H_9 cell proliferation (Supplementary Fig. 1c, g, h). T_H_9 cells from *Atg5-*deficient mice exhibit enhanced apoptosis compared to WT cells as detected by annexin V and 7-aminoactinomycin D (7-AAD) staining (Supplementary Fig. 1b, i, j). These results are in line with the work of Chi and co-workers^16^ who showed that Atg7-deficient Treg cells undergo increased cell death but increased proliferation.

We have tested whether autophagy deficiency affects T-cell survival and proliferation of T_H_1, T_H_2, T_H_9, T_H_17 and Treg cells in our settings. In line with previous studies^13, 16^, we noted upon autophagy blockade an increase in cell death and proliferation of all the CD4^+^ T-cell subsets studied following activation except for T_H_17 cells that proliferated like controls 72 h after differentiation initiation (Supplementary Fig. 1k, l, m). Importantly, although these observations did not translate into significant alterations of the overall cytokine accumulation during the course of T_H_1, T_H_2 and T_H_17 cell differentiation, we noted a fivefold increase in IL-9 secretion from autophagy-deficient T_H_9 cells (Fig. 1a). Overall, this indicates that autophagy blockade favours T_H_9 cell differentiation rather than inhibiting the differentiation of other effector T-cell subsets.

To further test whether *Atg5* affects T_H_9 cell differentiation, we knocked down *Atg5* expression in wild-type (WT) CD4 T cells using small interfering RNA (siRNA). In line with our results obtained using conditional deficient mice, we found that preventing Atg5 expression in T_H_9 cells enhanced their secretion of IL-9 (Fig. 1d and Supplementary Fig. 1n). Moreover, *Atg5* deficiency failed to enhance IL-9 expression in T_H_1, T_H_2, T_H_17 and Treg cells, suggesting that *Atg5* deficiency does not skew other effector or regulatory subsets towards a T_H_9 differentiation programme (Supplementary Fig. 1o). We have eventually investigated the involvement of Atg3 and LC3, both of which are required for autophagy^18^, in T_H_9 cell differentiation using *Atg3-* and *Map1lc3b-*deficient T cells. In line with our results obtained with *Atg5*-deficient mice, we found that CD4 T cells lacking *Atg3* or *Map1lc3b* featured enhanced ability to differentiate into T_H_9 cells compared to control cells (Fig. 1e, f), thereby demonstrating that deficiency of any key autophagy gene endows CD4 cells with augmented IL-9 secretion under T_H_9-skewing conditions. Overall, these results show that the expression of *Atg5* in differentiating T_H_9 cells cell-intrinsically and specifically represses their secretion of IL-9.

**Fig. 2:**
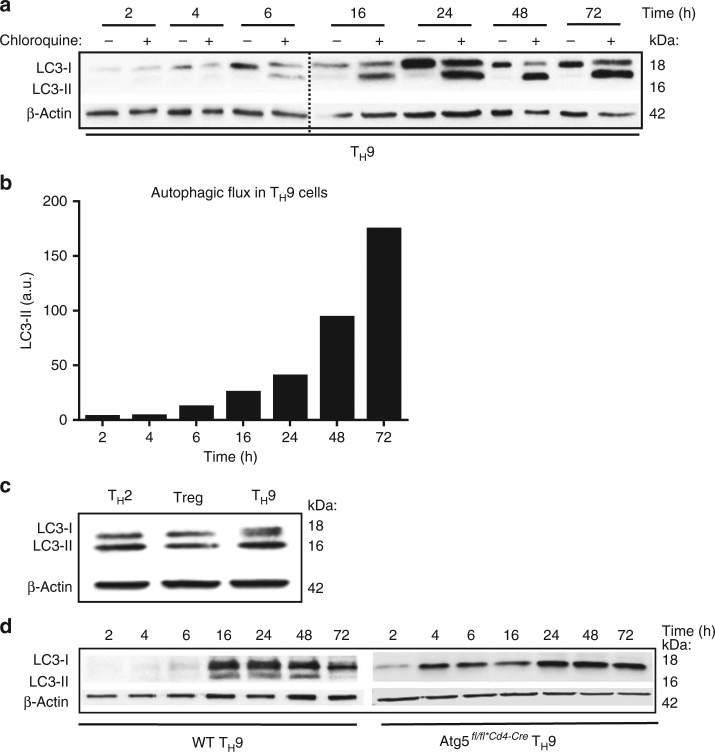
Autophagy is active during T_H_9 cell differentiation. **a** The conversion from endogenous LC3-I to LC3-II, the autophagosome marker, during T_H_9 cell differentiation was monitored by western blot in the presence and absence of chloroquine. The amounts of LC3-II on this immunoblot were used to determine the autophagic flux in T_H_9 cells. **b** The autophagic flux during T_H_9 cell differentiation was determined by calculating the differences in the amount of LC3-II between cells cultured with or without the lysosomal inhibitor chloroquine, thus representing the amount of LC3 that is delivered to lysosomes for degradation. **c** Immunoblot analysis of LC3-II expression in T_H_2, Treg and T_H_9 cells, differentiated for 3 days. **d** Western blot of LC3-II in Atg5-deficient cells compared to WT cells during T_H_9 cell differentiation

While *Atg5* is a key gene in the induction of autophagy^19^, it has been reported that *Atg5* could also signal independently of autophagy^20^. Thus, to test further the putative involvement of autophagy during T_H_9 cell differentiation, we next assessed the induction of autophagy in differentiating T_H_9 cells upon in vitro differentiation. Because the most reliable indicator of autophagic activity is the autophagic flux defined by the whole process of autophagosome synthesis followed by delivery to and degradation within lysosomes, we have monitored the autophagic flux in differentiating T_H_9 cells. Since LC3-II is degraded in autophagolysosomes we monitored LC3 turnover to measure autophagic flux. For this, we have differentiated naive CD4 T cells into T_H_9 cells in the presence or not of chloroquine, which prevents lysosomal degradation^21, 22^, and assessed LC3-II expression representing the amount of LC3-II that is delivered to lysosomes for degradation. Our time-course experiment showed higher levels of LC3-II in differentiating T_H_9 cells treated with chloroquine compared to control cells at every time point, strongly suggesting that the full process of autophagy occurs in differentiating T_H_9 cells (Fig. 2a, b and Supplementary Fig. 2a). In addition, when comparing autophagy levels in T_H_ cells, we found that T_H_9 cells featured comparable levels of lipidated LC3 (LC3-II) to T_H_2 and Treg cells, in which the induction of autophagy was previously shown^16, 17^ (Fig. 2c and Supplementary Fig. 2b). In the absence of Atg5, LC3-II expression is impaired during T_H_9 cell differentiation (Fig. 2d and Supplementary Fig. 2c), showing that Atg5 deficiency completely blunts autophagy in T_H_9 cells, thus demonstrating its capital function in T_H_9 autophagy process. Altogether, these results indicate that the genetic prevention of autophagy enhances T_H_9 cell generation in vitro.

### Pharmacological modulation of autophagy affects T_H_9 cells

**Fig. 3:**
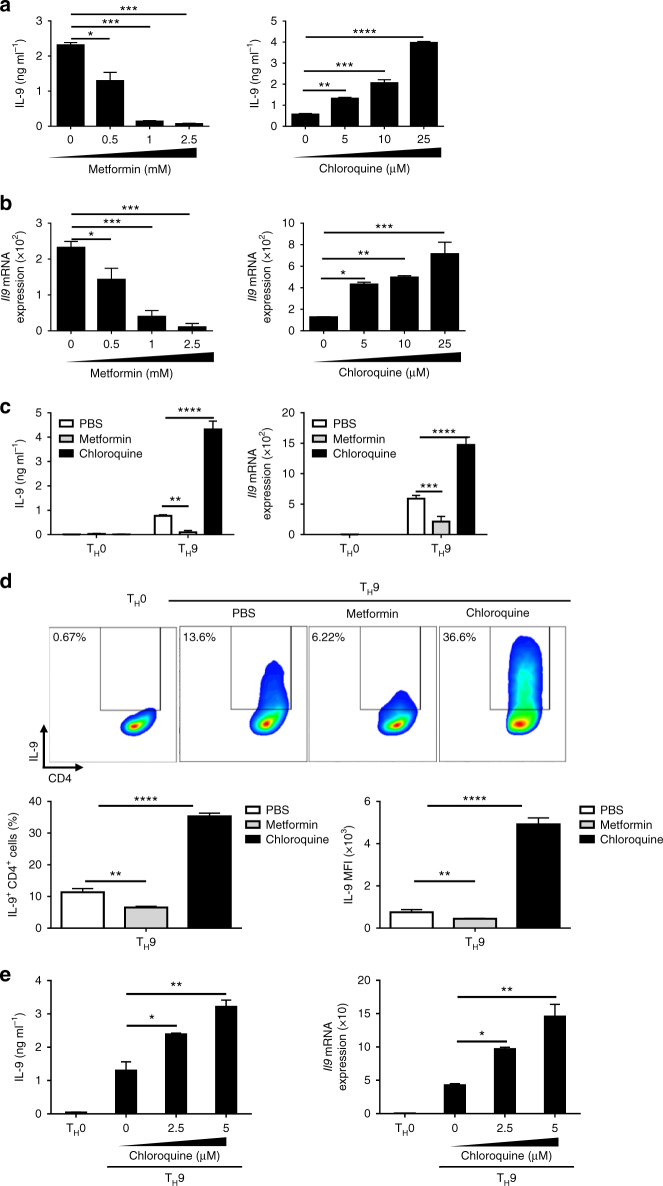
Mouse and human T_H_9 cell differentiation can be pharmacologically altered through modulation of autophagy. **a**, **b** Wild-type mouse naive CD4^+^ T cells were differentiated into T_H_9 cells with increasing doses of metformin or chloroquine and then IL-9 secretion levels were determined by ELISA and qRT-PCR after a 3-day culture. qRT-PCR results were normalized to the expression of *Actb*. Data are representative of three experiments. Naive CD4^+^ T cells were isolated from IL-9-EGFP mice and differentiated into T_H_9 cells for 3 days in the presence of metformin (1 mM) or chloroquine (25 μM) and IL-9 expression was analysed by **c** ELISA, qRT-PCR and **d** flow cytometry. FACS plots are representative of three experiments. The frequencies and mean fluorescence intensity (MFI) of IL-9-expressing cells are depicted (*right panels*). **e** Naive human CD4^+^ T cells were cell-sorted from three healthy blood donors and differentiated into T_H_9 cells in the presence of increasing doses of chloroquine. IL-9 expression was then analysed by ELISA and qRT-PCR, and results were normalized to the expression of *Actb* and are presented relative to control T_H_0 cells. One representative experiment with one donor is shown. Data are representative of three experiments. Mean (+s.d.), NS, not significant with *P*>0.05; *P<0.05; **P<0.01; ***P<0.001; ****P<0.0001 two- way analysis of variance (ANOVA) test

Several clinically approved drugs such as metformin and chloroquine have been shown to regulate autophagy^23–25^. Because our results showed that differentiating T_H_9 cells undergo autophagy, which represses their IL-9 secretion, we hypothesized that drugs affecting autophagy would also modulate T_H_9 cell differentiation. To test this, we have differentiated T_H_9 cells in the presence or not of metformin and chloroquine, which respectively induce and block autophagy^23–25^. We found that metformin and chloroquine respectively prevented and enhanced T_H_9 cell differentiation in a dose-dependent manner (Fig. 3a, b). To track the effect of metformin and chloroquine on IL-9-expressing cells in real time and at the single cell level, we used IL-9-EGFP mice^26^. Differentiation of naive CD4 T cells obtained from IL-9-EGFP mice into T_H_9 cells in the presence of metformin decreased IL-9 secretion measured by ELISA and quantitative PCR (qPCR) (Fig. 3c) and both reduced the frequency and mean fluorescence intensity of IL-9-expressing cells 3 days following differentiation initiation (Fig. 3d). In line with our previous experiments, a similar series of studies performed with chloroquine yielded opposite results, confirming that autophagy blockade enhances T_H_9 cell differentiation (Fig. 3c, d).To test the possible effect of these drugs on proliferation and apoptosis, T_H_9 cells were stained with Ki67 or annexin V and 7-AAD. T_H_9 cells treated with chloroquine show a significant increase of apoptosis and a reduced proliferation compared to control cells (Supplementary Fig. 3). Metformin however did not affect cell death of differentiating T_H_9 cells but reduced their proliferation (Supplementary Fig. 3). Thus, chloroquine, despite its effects on T_H_9 cell proliferation and apoptosis, markedly enhances T_H_9 cell IL-9 secretion on a per-cell basis, ultimately resulting in enhanced IL-9 secretion from chloroquine-treated T_H_9 cells (Supplementary Fig. 3). This overall supports the fact that the chloroquine-driven blockade of autophagy in T_H_9 cells supports their IL-9 secretion.

We have eventually tested the effects of chloroquine on human T_H_9 cell differentiation. For this, human CD4 T cells were isolated from three different healthy blood donors and differentiated into T_H_9 cells in the presence or not of increasing doses of chloroquine. In line with our results obtained with mouse T cells, we found that chloroquine enhanced IL-9 secretion from human T_H_9 cells in a dose-dependent manner (Fig. 3e). Collectively, although these results do not rule out any autophagy-independent effects of metformin and chloroquine on T_H_9 cell survival and proliferation, they strongly suggest that T_H_9 cell differentiation can be pharmacologically altered through the modulation of autophagy.

### Autophagy blockade enhances PU.1 expression in T_H_9 cells

**Fig. 4:**
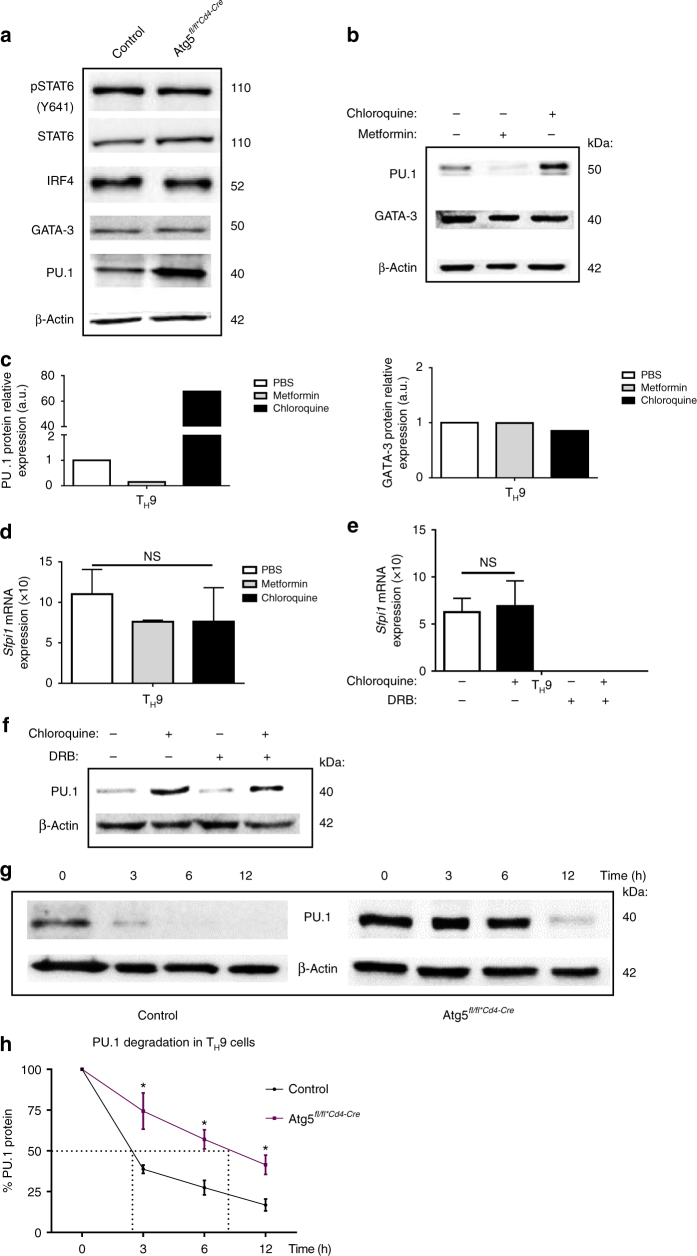
Autophagy modulates PU.1 protein stability in T_H_9 cells. **a** Naive CD4^+^ T cells were isolated from Atg5^fl/+*CD4-Cre^ and Atg5^fl/fl*CD4-Cre^mice and differentiated into T_H_9 cells for 24 h. Representative western blot of T_H_9 cell-related transcription factors in *Atg5*-deficient T_H_9 cells compared to controls. Data are representative of two experiments. **b** Naive CD4^+^ T cells were isolated and differentiated into T_H_9 cells for 24 h in the presence of metformin (1 mM) or chloroquine (25 µM). PU.1, GATA-3 and β-actin expression was analysed by western blot and its quantification is shown in **c**. **d** Same as in **b** and *Sfpi1* expression was determined by quantitative RT-PCR at 24 h from three experiments. **e** Naive CD4^+^ T cells were isolated and differentiated into T_H_9 cells for 16 h in the presence or absence of chloroquine (25 μM). Then, cells were treated with 25 μg ml^−1^ of DRB, an inhibitor of transcription, for 8 h. *Sfpi1* mRNA expression was analysed by qRT-PCR at 0 and 8 h of DRB treatment and **f** PU.1 expression was analysed by western blot at 8 h of DRB treatment. Experiments performed twice. All qRT-PCR results were normalized to the expression of *Actb* (mean+s.d.), NS, not significant, analysis of variance (ANOVA) test. **g** Naive CD4^+^ T cells were isolated from Atg5^fl/+*CD4-Cre^ and Atg5^fl/fl*CD4-Cre^ mice and differentiated into T_H_9 cells. PU.1 protein expression was assessed by western blot after being treated with 25 μg ml^−1^ cycloheximide to inhibit protein synthesis for 3, 6 and 12 h. PU.1 protein expression is plotted in **h** as a percentage of total PU.1 protein at time zero, and reflects the values obtained from western blot shown. The western blot shown is representative of two independent experiments. Mean (+s.d.), *P<0.05 two-way ANOVA test

We next investigated the mechanism responsible for the modulation of T_H_9 cell functions by autophagy. Because autophagy deficiency was shown to alter the function of Treg cells through the induction of glycolysis^16^, we first monitored the phosphorylation of ribosomal protein S6 (pS6) and 4E-BP1, which denote the activation of the metabolic regulator mammalian target of rapamycin complex 1 (mTORC1)^27^, in differentiating control or Atg5fl/fl*CD4-CreTreg cells and T_H_9 cells. In contrast to Treg cells where we found enhanced phosphorylated pS6 and 4E-BP1 in the absence of *Atg5* as recently reported^16^, the phosphorylation levels of these two proteins were similar in T_H_9 cells independently of *Atg5* expression (Supplementary Fig. 4). Thus, contrary to Treg cells, there is no contribution of mTORC1 in the autophagy-driven modulation of T_H_9 cell functions. We next monitored the expression of different transcription factors involved in T_H_9 cell polarization in differentiating control or Atg5fl/fl*CD4-Cre cells. Although the expression of Stat6, pStat6, IRF-4 and GATA-3 are not affected by autophagy deficiency, we noted that the expression of PU.1 protein, the T_H_9 cell master transcription factor encoded by *Sfpi1* gene, was markedly enhanced in T_H_9 cells deficient for *Atg5* compared to *Atg5*-expressing controls (Fig. 4a and Supplementary Fig. 5a, b, c). Accordingly, naive CD4 T cells differentiated into T_H_9 cells in the presence of metformin or chloroquine respectively featured repressed or enhanced levels of PU.1 protein compared to controls (Fig. 4b, c and Supplementary Fig. 5d). Importantly, *Sfpi1* mRNA was unaffected by the presence of metformin or chloroquine during T_H_9 cell differentiation, suggesting a post-transcriptional effect of these drugs on PU.1 expression (Fig. 4d). Interestingly, the protein expression of the transcription factor GATA-3, which is essential for T_H_9 cell differentiation^28^, was unaffected by metformin or chloroquine, indicating that autophagy selectively affects PU.1 protein expression (Fig. 4b, c). Because *Sfpi1* mRNA levels did not significantly change prior to the corresponding protein, we hypothesized that autophagy regulates PU.1 expression post-transcriptionally. To test this, we differentiated naive WT CD4 T cells in the presence or not of chloroquine into T_H_9 cells for 16 h and subsequently inhibited transcription using 5,6-dichloro-1-β-d-ribofuranosylbenzimidazole (DRB) and assessed *Sfpi1* mRNA expression after 8 h. We found that, while DRB efficiently prevented *Sfpi1* gene expression, it failed to block the chloroquine-induced enhanced expression of PU.1 protein (Fig. 4e, f and Supplementary Fig. 5e), indicating that the blockade of autophagy favours PU.1 expression in differentiating T_H_9 cells independently of transcription. We then tested whether autophagy affected the stability of PU.1 protein in differentiating T_H_9 cells. For this, we differentiated control or Atg5fl/fl*CD4-Cre cells into T_H_9 cells for 16 h and subsequently blocked translation using cycloheximide and kinetically monitored PU.1 degradation by western blot. We found that the degradation of PU.1 was notably reduced in Atg5fl/fl*CD4-Cre cells compared to control cells, indicating that the absence of autophagy prolongs PU.1 protein half-life in T_H_9 cells. Indeed, the absence of Atg5 promotes PU.1 protein stability, enhancing its half-life from 3 to more than 6 h (Fig. 4g, h and Supplementary Fig. 5f). To investigate the subcellular localization of PU.1 protein during T_H_9 cell differentiation we performed a kinetic experiment in T_H_9 cells from WT and *Atg5*-deficient mice followed by a subcellular fractionation. The expression of PU.1 was then analysed in nuclear and cytoplasmic fractions by western blot. In WT T_H_9 cells, PU.1 is mainly nuclear with an important nuclear expression from 8 to 48 h of differentiation, according to its function as transcription factor and master regulator of the T_H_9 programme. Interestingly, in the absence of Atg5, PU.1 protein expression is higher in both fractions compared to WT cells (Supplementary Fig. 6). PU.1 can be easily detected in the nuclear and cytoplasmic fractions of the cells from 8 h of differentiation (Supplementary Fig. 6). Overall, these results demonstrate that preventing autophagy in T_H_9 cells leads to enhanced stability of the T_H_9 cell transcription factor PU.1.

### Selective autophagy targets PU.1 for degradation

**Fig. 5:**
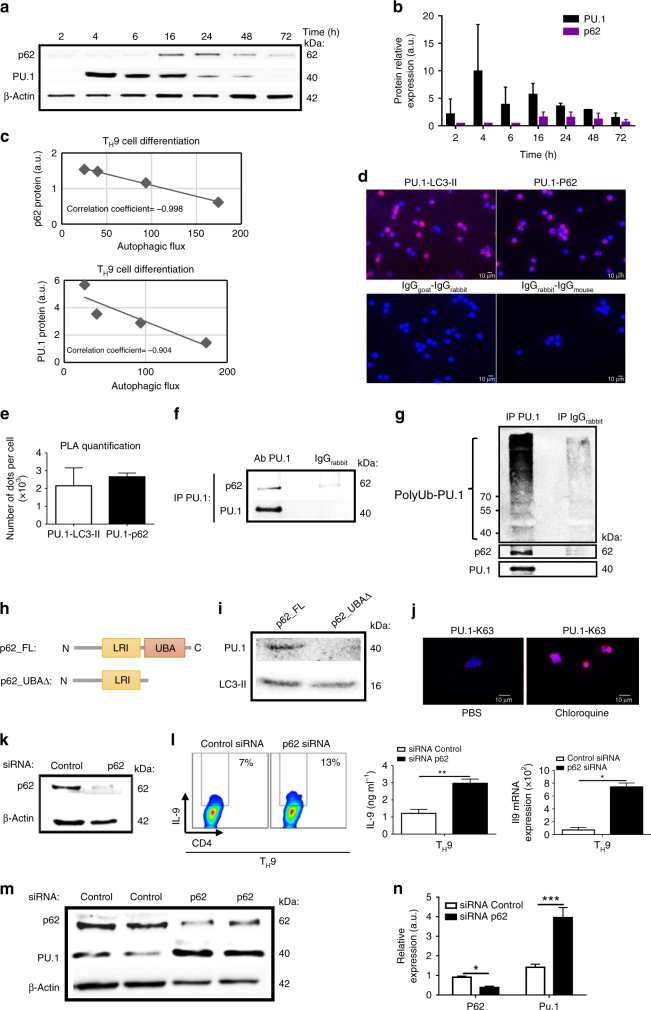
PU.1 protein is recruited by p62 and LC3-II and targeted for selective autophagy degradation in T_H_9 cells. **a** The expressions of p62 and PU.1 were analysed by western blot at different time points during T_H_9 cell differentiation. The western blot shown is representative of two independent experiments. **b** Quantification of the western blot shown in **a**. **c** Scatterplot showing the negative correlation between the amount of p62 or PU.1, determined by the western blot in **a** and the autophagic flux (determined in Fig. 2b). **d** Proximity ligation assay (PLA) showing the interaction between PU.1, LC3-II and p62 in T_H_9 cells, as well as control antibodies, after 24 h of differentiation. **e** PLA quantification: number of dots per cell out of 100 cells in two independent experiments. **f** Immunoblot corresponding to co-immunoprecipitation experiment showing interaction of endogenous PU.1 and p62 in chloroquine-treated T_H_9 cells (25 μM) after 24 h of differentiation. Shown is a typical experiment out of three. **g** Immunoblot corresponding to immunoprecipitation performed with anti-PU.1 antibody, followed by western blot detection using poly- and monoubiquitination antibody to examine the presence of poly-ubiquitinated PU.1 in chloroquine-treated T_H_9 cells (25 μM) after 24 h of differentiation. **h** Schematic representation of the p62 cDNA fragments used for pull-down experiments. **i** Synthetic full-length p62 (p62_FL) and p62 lacking the UBA domain (p62_UBA∆) proteins were generated. These two proteins were subsequently incubated with total extracts from T_H_9 cells differentiated for 24 h. The binding of endogenous PU.1 and LC3-II was tested in a pull-down assay and revealed using western blot analysis. Experiment performed twice. **j** PLA showing that in chloroquine-treated T_H_9 cells, PU.1 K63 ubiquitination can be detected after 24 h of differentiation. T_H_9 cells transfected with control siRNA or p62 siRNA. **k** p62 inhibition was assessed by western blot 48 h after transfection. **l** IL-9 expression was analysed by flow cytometry, ELISA and qRT-PCR after 72 h of differentiation. Shown is a typical experiment out of three. *P<0.05; **P<0.01 unpaired Student’s *t*-test. **m**, **n** p62 inhibition and PU.1 protein expression were assessed by western blot 48 h after transfection and quantified. Mean (+s.d.). Shown is a typical experiment out of three. *P<0.05; ***P<0.001 two-way analysis of variance (ANOVA) test

P62 protein, a selective autophagy adaptor, is removed from the cytoplasm upon autophagy^6, 29, 30^. This process is responsible for the selective degradation of proteins that bind to p62^6, 29, 30^. Because our observations indicated a selective degradation of PU.1 in T_H_9 cells, we have interrogated the contribution of p62-dependent selective autophagy in T_H_9 cell differentiation. For this, we first monitored p62 and PU.1 expression during the course of T_H_9 cell differentiation. Western blot analysis showed that p62 expression was induced in T_H_9 cells and peaked 16 h after the initiation of differentiation (Figs. 5a, b and Supplementary Fig. 7). As a selective autophagy adaptor, p62 is known to be degraded through autophagy^6, 29, 30^. Accordingly, kinetic monitoring of the total cellular expression levels of p62 inversely correlates with the autophagic activity during the course of T_H_9 cell differentiation (Fig. 5c), thereby suggesting that in T_H_9 cells p62 is also degraded through autophagy.

Because PU.1 is selectively degraded during T_H_9 differentiation, we next tested the putative interaction between PU.1, LC3-II and p62 in T_H_9 cells. For this, we first used a proximity ligation assay (PLA). We found that PU.1 interacts with LC3-II and p62 in T_H_9 cells, whereas no PLA foci were detected with control antibodies (Fig. 5d, e). Moreover, we also checked by PLA if other T_H_9 transcription factors are recruited by p62 and found no interaction for Stat6 or GATA-3 and a weak interaction for IRF-4 compared to PU.1 suggesting that, in T_H_9 cells, PU.1 is preferentially recruited by p62 (Supplementary Fig. 8a, b). Immunoprecipitation experiments confirmed the formation of a PU.1-p62 complex in T_H_9 cells treated in the presence of chloroquine (Fig. 5f and Supplementary Fig. 9a). Importantly, we also found that PU.1 was polyubiquitinated (Fig. 5g and Supplementary Fig. 9b), which is an essential mark to target a protein for selective autophagy degradation. We next analysed the PU.1 complexes in T_H_9 cells treated with chloroquine by mass spectrometry. As previously described^31^, PU.1 interacts with other transcription factors, mRNA splicing factors and RNA binding proteins (Table 1 and Supplementary Fig. 8c), showing that PU.1 is part of different complexes, and hence suggesting a variety of functions for this transcription factor in T_H_9 cells. Importantly, mass spectrometry analysis of PU.1 complexes revealed that p62 interacts with PU.1 (Table 2). p62 is known to bind to polyubiquitinated protein cargos via an ubiquitin-associated (UBA) domain^7^, and to autophagy machinery via a LC3-interacting region (LIR), thus making a bridge between polyubiquitinated cargos and autophagosomes. To test whether p62 interacts with PU.1 through its UBA domain we used a coupled transcription/translation in vitro system to generate p62 proteins labelled with biotinylated lysine. We synthesized both full-length p62 (p62_FL) and p62 lacking the UBA domain (p62_UBA∆). These two proteins were subsequently incubated with total extracts from T_H_9 cells differentiated for 24 h. The binding of endogenous PU.1 was tested in a pull-down assay and revealed using western blot analysis. Importantly, we found that UBA truncation significantly impaired p62 ability to recruit PU.1 without affecting its interaction with LC3-II (Fig. 5h, i and Supplementary Fig. 9c). Moreover, mass spectrometry analysis also reveals that PU.1 interacts with different K63 E3 ubiquitin ligases such as Ubr5, Hectd1 and Wwp2 (Table 2). As ubiquitination can target proteins for selective autophagy-dependent degradation with K63 ubiquitination^32^, we have assessed PU.1 ubiquitination by PLA and found enhanced PU.1 K63 ubiquitination in T_H_9 cells treated with chloroquine, suggesting that PU.1 is preferentially degraded through autophagy in T_H_9 cells (Fig. 5j). In line with this result, pharmacological inhibition of the proteasome with MG132, a proteasome inhibitor, failed to enhance PU.1 expression in differentiating T_H_9 cells, confirming that PU.1 degradation in T_H_9 cells is primarily driven through autophagy (Supplementary Fig. 10). To test the relevance of p62-dependent degradation of PU.1 in T_H_9 cell differentiation, we silenced p62 expression in differentiating T_H_9 cells with a siRNA preventing p62 expression. In line with our hypotheses, we found that p62 inhibition shown by western blot enhanced T_H_9 cell differentiation as illustrated by enhanced IL-9 secretion (Fig. 5k, l and Supplementary Fig. 11a, b). Importantly, p62 inhibition led to increased PU.1 expression (Fig. 5m, n and Supplementary Fig. 11c). Altogether, these findings indicate that the p62-dependent degradation of the PU.1 transcription factor through selective autophagy represses T_H_9 cell differentiation.

**Table 1:** Characterization of PU.1 complexes in T_H_9 cells

**Transcription factor**	**mRNA splicing factor**	**Translation factor**
Maff	Lsm3	Ddx54
Med12l	Snrnp70	Clic4
Zfp648	Wdr62	Gsto1
Stat1	Rbm22	
Trappc2	Srsf11	
Pdlim5		
Pdlim1		
Zbtb25		
Naca		
Rsf1		
Nr3c1		
Cnot2		
Psip1		
Crtc3		
Gtf2e1		
Stat4		
Ncor1		
Lrrfip1		
Tbp		
Ctcf		
Ddi1		
Lima1		
Zyx		
Trip4		
Gtf2f1		
p300		
Stat3		

Mass spectrometry analysis of PU.1 complexes in T_H_9 cells treated with chloroquine (25 μM) for 24 h. The table shows a short list of PU.1 partners that belong to transcription, splicing and translation machineries

**Table 2:** PU.1 complexes are associated with selective autophagy machinery

**Gene symbol**	**Peptide**	**XCorr**	Δ**Corr**
Ube2n	K.LELFLPEEYPM*AAPK.V	2.811	0.248
Ubr5	K.NSLEDLTAEDFR.L	3.303	0.476
Hectd1	R.DLVDKGGDIFLDQLAR.L	3.046	0.135
Wwp2	R.LYIIM*R.G	2.13	0.105
Huwe1	R.LLSLISIALPENK.V	3.185	0.448
Mib1	R.AVHHAAFGDEGAVIEVLHR.G	2.922	0.207
Ube2o	K.SGYPDIGFPLFPLSK.G	2.231	0.392
Trim33	K.SLLQQLENVTK.E	2.823	0.27
Rnf138	R.ALDLENIM*R.R	2.362	0.145
Sqstm1	K.NYDIGAALDTIQYSK.H	3.524	0.503

Mass spectrometry analysis of PU.1 complexes in T_H_9 cells treated with chloroquine (25 μM) for 24 h. Table showing some PU.1 partners such as p62 (Sqstm1) and different E3 ubiquitin ligases, the corresponding peptides as well as their associated XCorr (cross correlation) and ∆Corr (delta correlation) scores

### Autophagy blockade enhances T_H_9 cell anticancer functions

**Fig. 6:**
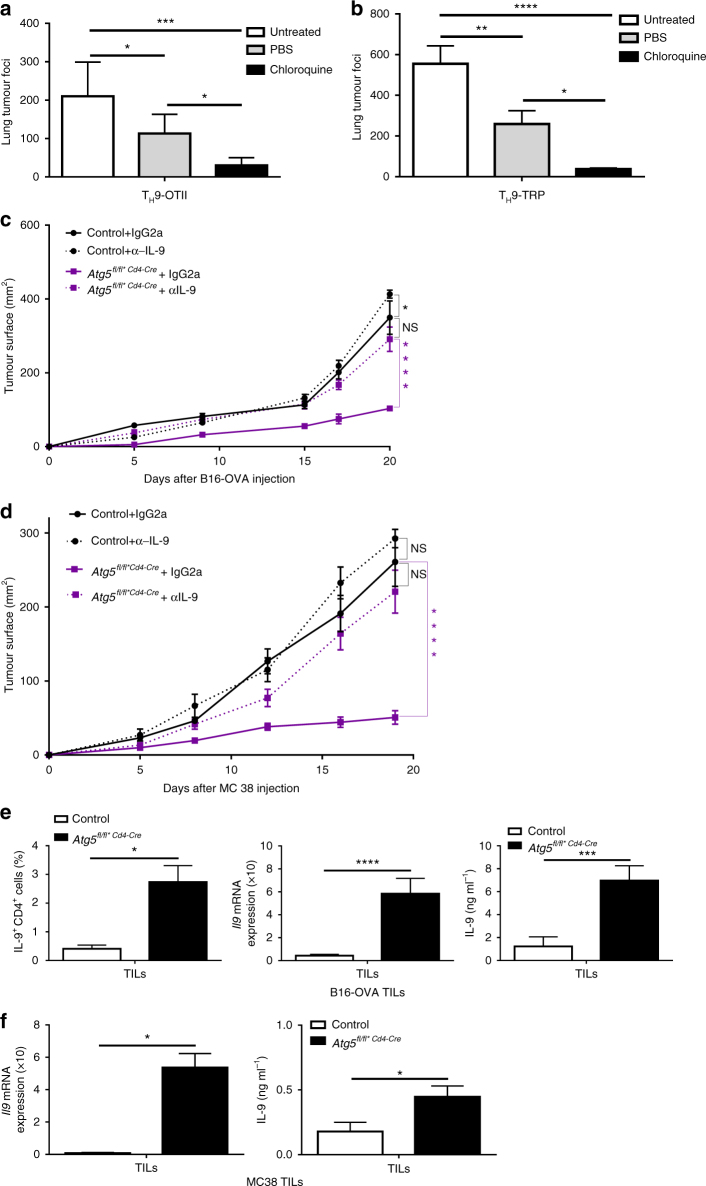
Pharmacological and genetic blockade of autophagy in T_H_9 cells enhance their IL-9-dependent antitumour functions in vivo. **a** WT mice were injected i.v. with 0.5×10^6^ B16-OVA melanoma cells and effector 2×10^6^ OT-II T_H_9 cells differentiated with or without chloroquine, and mean numbers of lung tumour foci are depicted (mean+s.d., 5 mice per group, 3 independent experiments). **b** WT mice were injected i.v. with 0.5×10^6^ B16 melanoma cells and effector 0.1×10^6^ TRP T_H_9 cells differentiated with or without chloroquine, and mean numbers of lung tumour foci are depicted (mean+s.d., 5 mice per group, 2 independent experiments). **c**, **d** Tumour growth (mean+s.e.m.) monitored three times a week, in B16-OVA or MC38 tumour-bearing control and Atg5^fl/fl*CD4-Cre^ mice that received control mouse IgG2a or anti-IL-9 neutralizing antibodies (10 mg per kg body weight) at days −1 and 0 and then twice a week for 20 days (mean+s.e.m.) **e** TILs of B16-OVA tumour-bearing control and Atg5^fl/fl*CD4-Cre^mice isolated at day 20 after tumour cell injection and stimulated with ovalbumin peptides OVA_323–339_ for 24 h. IL-9 expression from OVA_323–339_ CD4 stimulated TILs analysed by qPCR, ELISA and FACS. **f** MC38 TILs were stimulated with 50 ng ml^−1^ of PMA and 1 μg ml^−1^ of ionomycin for 24 h. IL-9 mRNA expression was analysed by qPCR and ELISA (mean+s.d., 5 mice per group, 2 independent experiments). NS, not significant with *P*>0.05; *P<0.05; **P<0.01; ***P<0.001; ****P<0.0001. **a**, **b** One-way analysis of variance (ANOVA) test; **c**, **d** two-way ANOVA test; **e**, **f** unpaired Student’s *t*-test

We and others have shown that T_H_9 cells harbour superior anticancer properties compared to those of T_H_1 or T_H_17 cells upon adoptive transfer in vivo^33, 34^. Because we found that autophagy blockade can enhance T_H_9 cell differentiation, we have examined the ability of T_H_9 cells differentiated in conditions preventing autophagy to suppress melanoma tumour growth in vivo. For this, B16-OVA cells (ovalbumin (OVA)-transfected B16F10 melanoma cancer cells) were injected intravenously (i.v.) into C57BL/6 mice along with CD4 T cells isolated from OT-II mice, which have a TCR specific for the OVA peptide (323–339) presented by major histocompatibility complex class II molecules, treated ex vivo with chloroquine and polarized into T_H_9 cells. The enumeration of the number of lung tumour foci after 14 days revealed that chloroquine-treated T_H_9 cells featured enhanced anticancer effects over control T_H_9 cells (Fig. 6a). In addition, by using CD4 T cells from TCR transgenic mice recognizing tyrosinase-related protein 1 (TRP-1)^35^, a melanocyte differentiation antigen naturally expressed by B16F10 melanoma cells, we also found that the anticancer activity of T_H_9 cells was similarly enhanced by chloroquine in the non-OVA B16F10 tumour model (Fig. 6b). To further investigate the links between T_H_9 cells and autophagy in a cancer setting, we implanted B16-OVA tumour cells subcutaneously (s.c.) into control or Atg5fl/fl*CD4-Cremice. We found that mice with selective *Atg5* deficiency in CD4 T cells featured reduced tumour growth over controls (Fig. 6c). Importantly, anti-IL-9 antibody treatment abrogated the beneficial effect of autophagy deficiency in CD4 T cells, proving that the anticancer effect is driven by enhanced T_H_9 cell function (Fig. 6c). Similar results were noted in the MC38 colon adenocarcinoma tumour model (Fig. 6d). By analysing the B16-OVA tumour infiltrate, we found enhanced frequency of IL-9-producing CD4 tumour-infiltrating lymphocytes (TILs). In vitro stimulation of TILs with OVA_323–339_ peptide led to marked increase of IL-9 from stimulated CD4 TILs, as detected by ELISA and qPCR (Fig. 6e). IL-9 has been shown to favour CD8 T-cell activation in a model of pulmonary melanoma in mice^36^. Accordingly, tumour infiltrate analysis showed enhanced frequency of IFN-γ-producing CD8 TILs in our system. Moreover, in vitro stimulation of TILs with OVA_257–264_ peptide also led to marked increase of IFN-γ from stimulated CD8 TILs as detected by ELISA and qPCR (Supplementary Fig. 12a). Finally, we obtained similar results with MC38 TILs from control or Atg5fl/fl*CD4-Cre mice (Fig. 6f and Supplementary Fig. 12b). Altogether, these results show that the suppression of autophagy in T_H_9 cells increases their ability to induce anticancer immune responses in vivo.

## Discussion

In this study we show that induction of autophagy in effector CD4 T cells selectively represses T_H_9 cell differentiation through a p62-dependent degradation of the T_H_9 cell master transcription factor PU.1. Accordingly, CD4 T cells lacking Atg3, Atg5 or LC3-II feature increased IL-9 secretion relative to control cells upon differentiation into T_H_9 cells. Moreover, pharmacological blockade of autophagy using chloroquine enhances both mouse and human T_H_9 cell differentiation. During T_H_9 cell differentiation, the T_H_9 cell master transcription factor PU.1 is K63-ubiquitinated and recruited by p62, which targets PU.1 for degradation through selective autophagy. Finally, genetic or pharmacological blockade of autophagy in T_H_9 cells not only enhances their anticancer functions in an IL-9-dependent manner against melanoma upon adoptive transfer but also in vivo in both established colon cancer and melanoma.

T_H_9 cells were initially coined by Kuchroo and Stockinger laboratories as proinflammatory cells that contribute to tissue inflammation and defence against parasites^28, 37^. Subsequently, this CD4 T-cell subset has gained further interest because of its potent anticancer properties, especially upon adoptive transfer into melanoma-bearing mice^33, 34, 38, 39^. However, the regulation of T_H_9 cell development in the tumour microenvironment is still incompletely understood. We here demonstrate the biological importance of the regulation of T_H_9 cells by autophagy in tumours. Our results suggest that in the tumour microenvironment T_H_9 cells undergo increased autophagy leading to decreased IL-9 expression. While the tumour-derived factors affecting T_H_9 cell induction remain to be fully characterized, our findings may begin to explain the low abundance of T_H_9 cells in tumours and why they fail to drive complete tumour clearance in the absence of additional therapeutic intervention.

PU.1 is an ETS family transcription factor that is induced by transforming growth factor-β (TGF-β) during T_H_9 cell differentiation and is essential for T_H_9 cell generation as illustrated by the inability of PU.1-deficient CD4^+^ T cells to produce IL-9 upon T_H_9 cell differentiation^40^. Interestingly, previous findings indicated that PU.1 selectively regulated IL-9 production in differentiating T_H_9 cells^40^. Our findings accordingly show that during T_H_9 cell differentiation the increased PU.1 protein level upon genetic or pharmacological inhibition of its degradation by autophagy enhances T_H_9 cell-derived IL-9, but not IL-10 or IL-21, secretion. Protein inclusions observed in the animal cells with defective autophagy are enriched in ubiquitin^22^. We similarly observed an accumulation of ubiquitinated PU.1 protein in T_H_9 cells treated with chloroquine. We also found that, through its UBA domain, p62 is able to bind ubiquitinated PU.1 and interacts with LC3-II. Our work thus suggests that PU.1 is selectively degraded during T_H_9 cell polarization through autophagy. While these results may contrast with previous studies indicating that PU.1 has a PEST domain and could thus be degraded by the proteasome, it is noteworthy that truncation of the PU.1 PEST domain did not lead to protein stabilization^41^, and treatments with proteasome inhibitors such as lactacystin only partially inhibited PU.1 degradation in F5-5 erythroblasts^42^. Eventually, we found that the differentiation of T_H_9 cells upon proteasome inhibition with MG132 failed to stabilize PU.1 protein, suggesting that in T_H_9 cells the degradation of PU.1 is predominantly driven through selective autophagy.

Chi and co-workers^16^ recently showed that autophagy affects the stability of Treg cells by coupling environmental cues and metabolic homeostasis. Here we show that autophagy can also impair T_H_9 cell stability but through an mTORC1-independent mechanism. Indeed, Atg5-deficient T_H_9 cells do not show increased mTORC1 activity. This observation suggests that autophagy can regulate CD4 T-cell biology in many ways that remain largely misunderstood. For instance, Kabat et al.^43^ elegantly reported how autophagy differentially regulates Treg and T_H_2 cells to control intestinal inflammation. They found that while Atg16l1-deficient Treg cells showed significant decreased survival compared to WT Treg cells, Atg16l1-deficient T_H_2 cells have increased survival. The authors attribute this observation to the sensitivity of T cells, which tend to shift from glycolysis when autophagy is affected to environmental perturbations in the autophagy pathway. Autophagy deficiency in Treg cells upregulates metabolic regulators and glycolysis, thus impairing Treg cell survival^16^. In contrast to Treg cells, T_H_2 cells were shown to display an increased glycolytic rate^44^. This enhanced glycolytic metabolism used by T_H_2 cells makes them resistant to the metabolic changes triggered by autophagy deficiency. One important observation is that, like in T_H_2 cells, GATA-3 expression is essential for T_H_9 but not for Treg cell differentiation^28^. Meanwhile, expression of *Gata3* is linked to induction of glycolysis after TCR activation in T cells and orchestrates essential metabolic adaptations enforced by autophagy deficiency^45^. Thus, differential expression of factors such as GATA-3 may lead to different metabolic responses in the absence of autophagy in CD4 T cells and explain why T_H_9 cells behave like T_H_2 cells regarding glycolysis.

Autophagy is essential to control inflammation^46–48^ this is exemplified by the ability of the amino acid sensor GCN2 to control gut inflammation through autophagy induction in CD11c antigen-presenting cells as well as epithelial cells, resulting in the repression of T_H_17 cell responses^49^. In humans, polymorphisms in several autophagy-related genes, including IRGM, LRRK2, SMURF1 and ATG16L1, leading to autophagy deficiency are linked to inflammatory bowel disease (IBD) susceptibility^50^. Crohn’s disease and ulcerative colitis (UC) are the two most common forms of IBD. Strikingly, recent studies have shown an important function of T_H_9 cells in driving UC. T_H_9 cells expressing PU.1 were found in the gut mucosa of patients with UC as well as target cells expressing IL-9 receptor in the intestinal epithelium of IBD patients, suggesting that T_H_9 cells are relevant for the pathogenesis of IBD in humans^51, 52^. Moreover, T_H_9 transcription factor PU.1 and IL-9 expression were shown to be crucial for the development of experimental colitis in two different mouse models^28, 53^. Our findings that autophagy induction in T_H_9 cells reduces PU.1 stability and represses T_H_9 cell differentiation therefore suggest that autophagy prevents Th9 cell differentiation to limit inflammation.

Given the strong effector functions harboured by T_H_9 cells, we believe that the identification of autophagy as a factor dictating IL-9 secretion from T_H_9 cells provides significant insights to design new therapies not only against cancer but also to alleviate the course of allergic and autoimmune diseases. Moreover, these findings may have implications for the consideration of chloroquine for antitumour immunotherapy. Given the strong antitumour activity of T_H_9 cells in vivo, our findings could also be exploited for the adoptive T-cell therapy of cancer.

## Methods

### Mouse strains

All animals were bred and maintained according to both the FELASA (Federation of European Laboratory Animal Science Associations) and the Animal Experimental Ethics Committee Guidelines (University of Burgundy, Dijon, France and Zhejiang University, Hangzhou, China). Animals were used between 6 and 12 weeks of age. Female C57BL/6 mice were purchased from Charles River Laboratories (France) and Joint Ventures Slipper BK Experimental Animal (Shanghai, China). Mice were housed in specific pathogen-free facilities, and the experimental protocols were approved by the Animal Care and Use Committee of School of Medicine, Zhejiang University (Hangzhou, China) and of University of Burgundy (Dijon, France). Whenever required, animals were euthanized by CO_2_ exposure. TCR transgenic mice in which CD4^+^ T cells recognize OVA-derived antigen (OT-II) were provided by the CDTA (Cryopréservation, Distribution, Typage et Archivage animal, Orléans, France). TCR transgenic mice in which CD4^+^ T cells recognize the TRP-1 antigen^35^, CD4-Cre mice and Atg5^fl/fl^ mice were respectively purchased from JAX (stock numbers 8684 and 17336) and RIKEN (RBRC02975). Floxed Atg3 mice and ER-Cre mice were kindly provided by P. Youwen He (Duke University). Atg3^fl/fl^ mice were bred with ER-Cre mice to generate Atg3^fl/fl^ER-Cre mice. To induce Atg3 deficiency Atg3^fl/fl^ ER-Cre mice were intraperitoneally (i.p.) injected with 100 μg tamoxifen (Sigma) for 3 consecutive days. LC3-B-deficient mice were kindly provided by Professor Zhihua Chen (Zhejiang University). IL-9-EGFP mice^26^ were provided by Professor Richard Flavell. All transgenic mice used were on a C57BL/6 background and were age-matched with WT controls for experiments. Male mice were used for experiments using mice having CD4 T cells recognizing the TRP-1 antigen. Otherwise, female mice were used.

### Tumour growth experiments

B16F10, B16-OVA melanoma and MC38 colon adenocarcinoma cells were cultured at 37 °C under 5% CO_2_ in RPMI-1640 with glutamax-1 (Lonza) supplemented with 10% (vol/vol) fetal calf serum (Lonza), 1% penicillin, streptomycin, amphotericin B (Gibco), 4 mM HEPES (Gibco) and 1 mM sodium pyruvate (Gibco). B16-OVA and MC38 cells were kindly provided by Dr Rosenblatt (University of Miami Miller School of Medicine, Miami, FL, USA) and Professor Smyth (QIMR Berghofer Medical Research Institute, Brisbane, Australia), respectively. B16F10 cells were obtained from ATCC. All cells were routinely tested for mycoplasma contamination using Mycoalert Mycoplasma Detection Kit (Lonza, LT07-318) and found negative. 1×5.10^5^ B16-OVA or 1×10^6^ MC38 cells were injected s.c. into mice. In vivo IL-9 neutralization was achieved by i.p. injection (10 mg per kg body weight) of anti-IL-9 neutralizing antibody (MM9C1; BioXcell) on days −1 and 0 and twice a week following tumour implantation. As control we used control mouse IgG2a (in Vivo mAb Mouse IgG2a Isotype Control BioXcell BE0085). Tumour size was measured three times a week during 20 days following tumour implantation using a calliper. Animals with ulcerated tumours or with tumours above 300 mm^2^ in size were killed for ethical reasons. TILs were prepared by enzyme digestion with 1 mg ml^−1^ collagenase (Roche), 0.5 mg ml^−1^ DNase I and 25 μg ml^−1^ hyaluronidase (Sigma-Aldrich) at 37 °C for 30 min, followed by Percoll (GE Healthcare) gradient purification and analysed by flow cytometry, ELISA and qRT-PCR after stimulation with OVA (323–339) or OVA (257–264) peptides (Proimmune) at a final concentration of 10 µg ml^−1^ and 20 µg ml^−1^ respectively for the B16-OVA tumour model. For the MC38 in vivo model, TILs were stimulated with 50 ng ml^−1^ of phorbol 12-myristate 13-acetate (PMA) (Sigma-Aldrich) and 1 μg ml^−1^ of ionomycin (Sigma-Aldrich).

Alternatively, 5×10^5^ B16-OVA and 2×10^6^ effector OT-II T_H_9 cells differentiated in the presence or in the absence of chloroquine (20 μM) were injected i.v. into C57BL/6 mice. Lung tumour foci were enumerated after 14 days in a blinded fashion. Similarly, 5×10^5^ B16F10 and 5×10^4^ effector TRP-1 T_H_9 cells differentiated in the presence or in the absence of chloroquine (20 μM) were injected i.v. into mice. Lung tumour foci were enumerated after 14 days in a blinded fashion.

### In vitro T-cell differentiation

Naive CD4^+^ T cells (CD4^+^CD62L^hi^CD44^lo^) were obtained from spleens and lymph nodes of mice. Isolated naive T cells were routinely 98% pure. CD4^+^ T cells were purified from spleen and lymph nodes with anti-CD4 microbeads (Miltenyi Biotec), and then were further sorted as naive CD4^+^CD62L^hi^ T cells by flow cytometry with antibodies anti-CD4-FITC (RM4-4, BD Biosciences) and anti-CD62L-Alexa 700 (MEL-14, BD Biosciences). Isolated naive CD4^+^ T cells were stimulated with plate-bound antibodies against CD3 (145-2C11, 2 μg ml^−1^, BioXcell) and CD28 (PV-1, 2 μg ml^−1^, BioXcell) and polarized into effector CD4^+^ T lymphocyte subsets without cytokines (T_H_0 cells), or with IL-12 (20 ng ml^−1^) for T_H_1 cells, or with IL-4 (20 ng ml^−1^) for T_H_2 cells, or with TGF-β (2 ng ml^−1^) and IL-6 (25 ng ml^−1^) for T_H_17 cells, or with mouse TGF-β (2 ng ml^−1^) and IL-4 (20 ng ml^−1^) for T_H_9 cells. In some experiments, metformin or chloroquine were added (Sigma-Aldrich, France). Cells were classically harvested on day 3 (unless otherwise specified) for detection of cytokines by ELISA and RT-qPCR analysis. For human in vitro T-cell differentiation, naive T cells were sorted from healthy donors and stimulated with plate-bound antibodies against CD3 (5 μg ml^−1^, BioXcell) and CD28 (5 μg ml^−1^, BioLegend) and polarized into T_H_9 cells with TGF-β (10 ng ml^−1^) and IL-4 (5 ng ml^−1^) (R&D systems).

### siRNA transfection

Transient transfection of naive CD4^+^ T cells was performed in vitro using Lipofectamin RNAiMAX (Invitrogen, Carlsbad, CA, USA) according to the manufacturer’s instructions. 5×10^5^ cells were plated in 96-well plates, and siRNAs were transfected at 25 nM final concentration for 24 h. Then, 24 h after transfection, CD4^+^ T cells were stimulated with plate-bound anti-CD3 and anti-CD28 antibodies, differentiated into T_H_9 cells as described above and cultured for an additional 24 or 72 h before analysis with Silencer Select Predesigned siRNA specific for murine *Atg5* (ID: 161983, Ambion, Life Technologies) or murine *sqtsm1* (siGENOME Mouse Sqstm1 ((18412) D-047628-03-0020 GE Healthcare, Dharmacon) or Silencer Negative Control No. 1 (Sigma SIC001).

### Retroviral infection of CD4 T cells

Retrovirus preparation was performed in Plat-E cells. Plat-E cells were transfected with 7.5 μg pMX-IRES-GFP containing Cre gene, the media were replaced with fresh medium after 10 h and retrovirus supernatant was collected after additional 72 h.

Naive CD4 T cells from Atg5^fl/fl^ mice were stimulated with 2 μg ml^−1^ anti-CD3/CD28 overnight. Then, the retrovirus was added to the cells, followed by 4 h of incubation at room temperature and removal of the retrovirus. The CD4 T cells were cultured in different T helper cell-polarized conditions for 5 days.

### Measurement of cytokines

After 72 h of polarization, cell culture supernatants were assayed by ELISA for mouse IFN-γ, IL-4, IL-5, IL-13, IL-17 and IL-9 (BioLegend) according to the manufacturer’s protocol.

For intracellular staining, cells were cultured for 3 days, restimulated for 2 additional days and then stimulated for 4 h at 37 °C in culture medium containing PMA (50 ng ml^−1^; Sigma-Aldrich) and ionomycin (1 μg ml^−1^; Sigma-Aldrich). After staining for surface markers and 7-AAD, cells were fixed and permeabilized according to the manufacturer’s instructions (Cytofix/Cytoperm kit, BD Biosciences), and then stained for intracellular products. Monoclonal antibodies used for flow cytometry analyses were as follows: fluorescein isothiocyanate (FITC)-conjugated anti-CD4 (GK1.5, BD Biosciences, dilution 1:300), PC7-conjugated CD8 (BD Biosciences, dilution 1:300), allophycocyanin (APC7)-conjugated CD45 (BD Biosciences, dilution 1:300), Brilliant Violet (BV421)-conjugated Foxp3 (BD Biosciences, dilution 1:100), phycoerythrin (PE)-conjugated IFN-γ (BD Biosciences, dilution 1:300), PE-conjugated IL-4 (BD Biosciences, dilution 1:300), APC-conjugated anti-IL-9 (RM9A4, BioLegend, dilution 1:100) and APC-conjugated IL-17A (Pharmingen, dilution 1:300). All events were acquired by a BD LSR-II cytometer equipped with BD FACSDiva software (BD Biosciences) and data were analysed using FlowJo software (Tree Star, Ashland, OR, USA).

### Cell lysis

RIPA lysis for western blot (WB): purified naive T cells were differentiated into T_H_2, T_H_9 and Treg cells. Cell pellets were incubated in five volumes of RIPA buffer (50 mM Tris-HCl (pH 7.5), 150 mM NaCl, 1% NP-40, 0.5% Nadeoxycholate, 0.1% sodium dodecyl sulfate, 1 mM EDTA, protease inhibitors) for 45 min on ice. After centrifugation at 16,000×*g* for 15 min at 4 °C, the supernatant was recovered for WB analysis.

Low salt (LS) lysis for immunoprecipitation: purified naive T cells were differentiated for 24 h into T_H_9 cells in the presence or in the absence of chloroquine. Cell pellets were incubated in two volumes of LS buffer (10% glycerol, 20 mM Tris-HCl (pH 8), 0.2 mM EDTA, 0.1 M KCl, 0.5% NP-40, protease inhibitors) for 10 min. Centrifugation at 16,000×*g* for 15 min at 4 °C was used to recover the supernatant for immunoprecipitation analyses.

Protein concentration was assessed using the Bio-Rad DC protein assay kit. Proteins were then denaturated, loaded and separated on SDS-polyacrylamide gels and transferred on nitrocellulose membranes (Schleicher and Schuell). After blocking with 5% milk in phosphate-buffered saline and 0.1% Tween-20 (BST), membranes were incubated overnight with primary antibody diluted in BST washed and incubated for 1 h with secondary antibody diluted in BST/5% milk. After additional washes, membranes were incubated with luminol reagent (Santa Cruz Biotechnology, Heidelberg, Germany) and exposed to Bio-Rad molecular imager ChemiDoc.

### Immunoprecipitation

Total purified naive CD4 T cells were differentiated for 24 h into T_H_9 cells. Cell lysate was obtained by LS lysis as described above. Then, 1 ml of protein lysate (8 μg/μl) was pre-cleared by incubation with 100 μl of protein A/G beads (Invitrogen) on a wheel for 1 h at 4 °C. After 1 min of centrifugation at 400×*g* the lysate was recovered for the immunoprecipitation (20 ml of pre-clear lysate corresponding to the input was conserved at −20 °C). Next, 500 μl of the pre-cleared lysate was incubated with 10 μg of PU.1 antibody or control IgG rabbit antibody (Sigma) on a wheel for 2 h at 4 °C. A total of 50 μl protein A/G beads were added before incubating on a wheel for 1 h at 4 °C. Finally, the beads were collected by centrifugation, washed three times in LS buffer, and analysed by WB.

### Protein pull-down by biotinylated p62 proteins

RT reactions were performed using The SuperScript III First-Strand Synthesis System (Life Technologies) and total RNA from T_H_9 cells at 24 h of differentiation. PaltinumTaq DNA polymerase high fidelity (Invitrogen) was used for PCR amplification of desired fragments corresponding to full-length p62 (p62_FL) and p62 lacking the UBA domain (p62_UBA∆). All forward primers had a T7 promoter sequence: 5′-taatacgactcactatagggaga-3′ while reverse primer had a TAA stop codon. The primers were:

p62_FL: forward 5′-ataaaagctgggctctcggc-3′; reverse: 3′-tcacaatggtggagggtgctt-5′ and p62_UBA∆: forward 5′-atggcgtcgttcacggtgaa-3′; reverse: 3′-cttcagccctgtgggtcctt-5′.

Synthetic p62_FL and p62_UBA∆ proteins were then generated using TnT Quick Coupled Transcription/Translation Systems (Promega L1170) combined with biotinylated lysine (Transcend tRNA Promega L5061) according to the manufacturer’s instructions. These two proteins were subsequently incubated with total extracts from T_H_9 cells differentiated for 24 h for pull-down assay. For this we used the Pierce Pull-down Biotinylated-protein: protein interaction kit (Thermo Scientific 21115) according to the manufacturer’s instructions. The binding of endogenous PU.1 and LC3-II was revealed by western blots.

### Cell fractionation

1×10^6^ T_H_9 cells differentiated for 8, 16, 48 and 72 h were pelleted at 1,800 r.p.m. for 5 min. The pellet was resuspended in 50 µl of buffer A (10 mM KCl, 20 mM HEPES (pH 7.5), 1.5 mM MgCl_2_, 1 mM EDTA, 1 mM EGTA, 250 mM sucrose and protease inhibitors (Complete, Roche)) and after a quick vortex cells were kept on ice for 30 min. The mixture was then flushed a few times using 26G needle. Centrifugation at 1,000×*g* for 10 min at 4 °C allowed us to recover the supernatant containing the cytoplasmic fraction while the nuclear fraction was pelleted. Finally, the nuclear pellet was sonicated for 6 min (1-min ON and 1-min OFF) at high intensity using the Bioruptor sonicator.

### Mass spectrometry analysis

Purified naive T cells were differentiated into T_H_9 cells for 24 h, and endogenous PU.1 complex was immunopurified as described above. PU.1 complex was then separated on a 4 to 12% polyacrylamide gel (Invitrogen) and stained with coomassie from Bio-Rad (G-250 Stain) according to the manufacturer’s instructions. Mass spectrometry identification of proteins was carried out by R. Tomaino at Taplin Mass Spectometry Facility at Harvard Medical School (Boston, MA, USA).

### Proximase ligation assay

Purified naive T cells were differentiated into T_H_9 cells for 24 h. Cells were fixed with 4% paraformaldehyde for 15 min at room temperature and permeabilized with ice-cold MetOH for 10 min. After being blocked with a solution containing 0.5% bovine serum albumin in phosphate-buffered saline for 30 min at room temperature, the cells were incubated with the primary antibody overnight at 4 °C. Then, PLA was performed with Duolink In situ reagents according to the manufacturer’s instructions. Images were acquired using Zeiss Axio Imager M2 microscope.

### Antibodies

The antibodies were as follows: anti-APG5 (FL-275 Santa Cruz sc-33210, dilution 1:1,000) for WB, anti-LC3B (Sigma-Aldrich L7543, dilution 1:100) for WB and PLA, anti-p-Stat6 pY641 (BD Pharmingen 558241, dilution 1:1000) for WB, anti-Stat6 (Cell Signaling 9362, dilution 1:1000 for WB and 1:500 for PLA) for WB and PLA, anti-IRF4 (M-17 Santa Cruz sc-6059, dilution 1:1000 for WB and 1:500 for PLA) for WB and PLA, anti-GATA-3 (HG3-31 Santa Cruz sc-268, dilution 1:1000 for WB) for WB and anti-GATA-3 (H-48 Santa Cruz, dilution 1:500) for PLA, anti-PU.1 (T-21 Santa Cruz sc-352, 10 µg) for IP and (D-19 sc-5949, dilution 1:100) for PLA, anti-PU.1 (Cell Signaling 2266, dilution 1:100) for WB, anti-p62 (G962-C Progen, dilution 1:1000) for WB, mono- and polyubiquitinylated conjugates, mAb (FK2) HRP conjugate (Enzo BML-pw0150 dilution 1:1000) for WB, K48-linkage specific-polyubiquitin (D9D5 Cell Signaling 12805, dilution 1:1000) for PLA, K63-linkage specific-polyubiquitin (D7A11 Cell Signaling 12930, dilution 1:500) for PLA, anti-β-actin (Sigma-Aldrich, dilution 1:1000), Rabbit, Goat and Mouse IgG Isotype Control (Thermo Scientific, dilution 1:500 for PLA and 10 µg for IP) for IP and PLA, paxillin antibody B2 (Santa Cruz sc-365379, dilution 1:1000) and HDAC1 (Cell Signaling 2062, dilution 1:1000) for WB.

### Drug treatment

Chloroquine diphosphate (Sigma-Aldrich c6628) treatment: purified naive T cells were differentiated for 24 to 72 h into T_H_9 cells with chloroquine added at a final concentration of 25 μM (unless otherwise specified).

Metformin hydrochloride (Sigma-Aldrich PHR1084) treatment: purified naive T cells were differentiated for 24 to 72 h into T_H_9 cells with metformin added at a final concentration of 1 mM (unless otherwise specified).

DRB (Sigma-Aldrich D1916) treatment: purified naive T cells were differentiated for 16 h into T_H_9 cells and then 25 μg ml^−1^ of DRB was added for 8 h.

Cycloheximide (Sigma-Aldrich C 1988) treatment: purified naive T cells were differentiated into T_H_9 cells with cycloheximide added at a final concentration of 25 μg ml^−1^ during 0, 3, 6, 12 and 24 h.

MG132 (Sigma-Aldrich Z-Leu-Leu-Leu-al C2211) treatment: purified naive T cells were differentiated into T_H_9 cells with MG132 added at a final concentration of 2 mM during 24 h.

### Real-time quantitative PCR

Total RNA from T cells was extracted with TriReagent (Ambion), reverse transcribed using M-MLV Reverse Transcriptase (Invitrogen) and was analysed by RT-qPCR with the Sybr Green method according to the manufacturer’s instructions using the 7500 Fast Real Time PCR system (Applied Biosystems). Expression was normalized to the expression of mouse *Actb*. Primers designed to assess gene expression are as follows: *Actb* 5′-atggaggggaatacagccc-3′ and 5′-ttctttgcagctccttcgtt-3′; *Ifng* 5′-*gagctcattgaatgcttggc*-3′ and 5′-*gcgtcattgaatcacacctg*-3′; *Il4* 5′-*cgagctcactctctgtggtg*-3′ and 5′-*tgaacgaggtcacaggagaa*-3′; *Il5* 5′-*catttccacagtacccccac*-3′ and 5′-*gcaatgagacgatgaggctt*-3′; *Il9* 5′-aacagtccctccctgtagca-3′ and 5′-aaggatgatccaccgtcaaa-3′; *Il10* 5′-*tgtcaaattcattcatggcct*-3′ and 5′-*atcgatttctcccctgtgaa*-3′; *Il13; Il17a* 5′-tgagcttcccagatcacaga-3′ and 5′-tccagaaggccctcagacta-3′; *Il21* 5′-aaaacaggcaaaagctgcat-3′ and 5′-tgacattgttgaacagctgaaa-3′; *Il1β 5*′*-ggtcaaaggtttggaagcag-3*′ and 5′-*tgtgaaatgccaccttttga-3*′; *Stat6* 5′-tgcccggtctcacctaacta-3′ and 5′-ctggggtggtttcctcttg-3′; *Irf4* 5′-*caaagcacagagtcacctgg*-3′ and 5′-*tgcaagctctttgacacaca*-3′; *Gata3* 5′-aggatgtccctgctctcctt-3′ and 5′-gcctgcggactctaccataa-3′; *Sfpi1* 5′-*tgcagctctgtgaagtggtt*-3′ and 5′-*agcgatggagaaagccatag*-3′; *Foxp3* 5′-ctcgtctgaaggcagagtca-3′ and 5′-tggcagagaggtattgaggg-3′.

### Statistical analyses

Statistical analysis was performed using Prism software (Graph Pad software, La Jolla, CA, USA). Student’s *t*-test and analysis of variance test were used for statistical analyses.

### Data availability

All relevant data are available from the authors.

## Electronic supplementary material

Supplementary Information
