# Supplementary material for: Selective degradation of PU.1 during autophagy represses the differentiation and antitumour activity of TH9 cells
Source: Nat Commun. 2017 Sep 15;8:559. doi: 10.1038/s41467-017-00468-w (PMC5602674; doi:10.1038/s41467-017-00468-w)
Supplement: Supplementary file 1 — Supplementary Information [file 41467_2017_468_MOESM1_ESM.pdf]

Supplementary Fig. 1

a

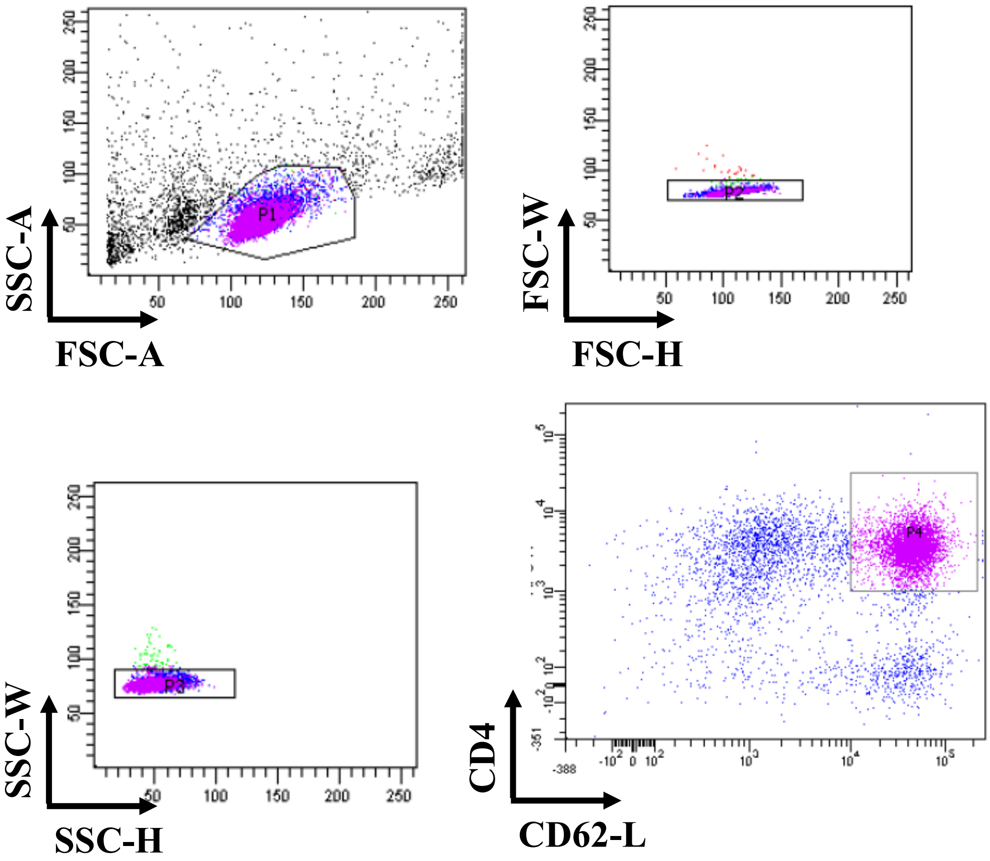

b

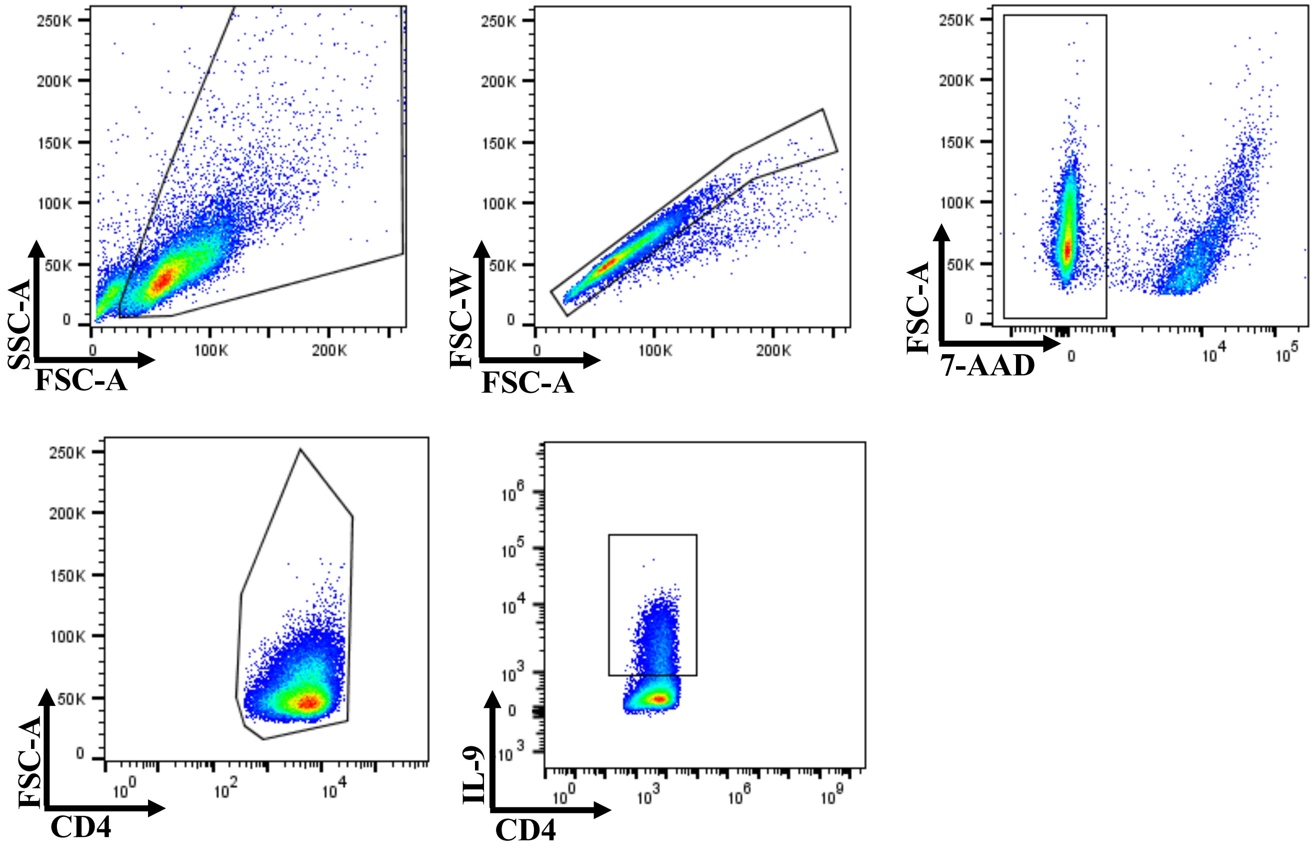

c

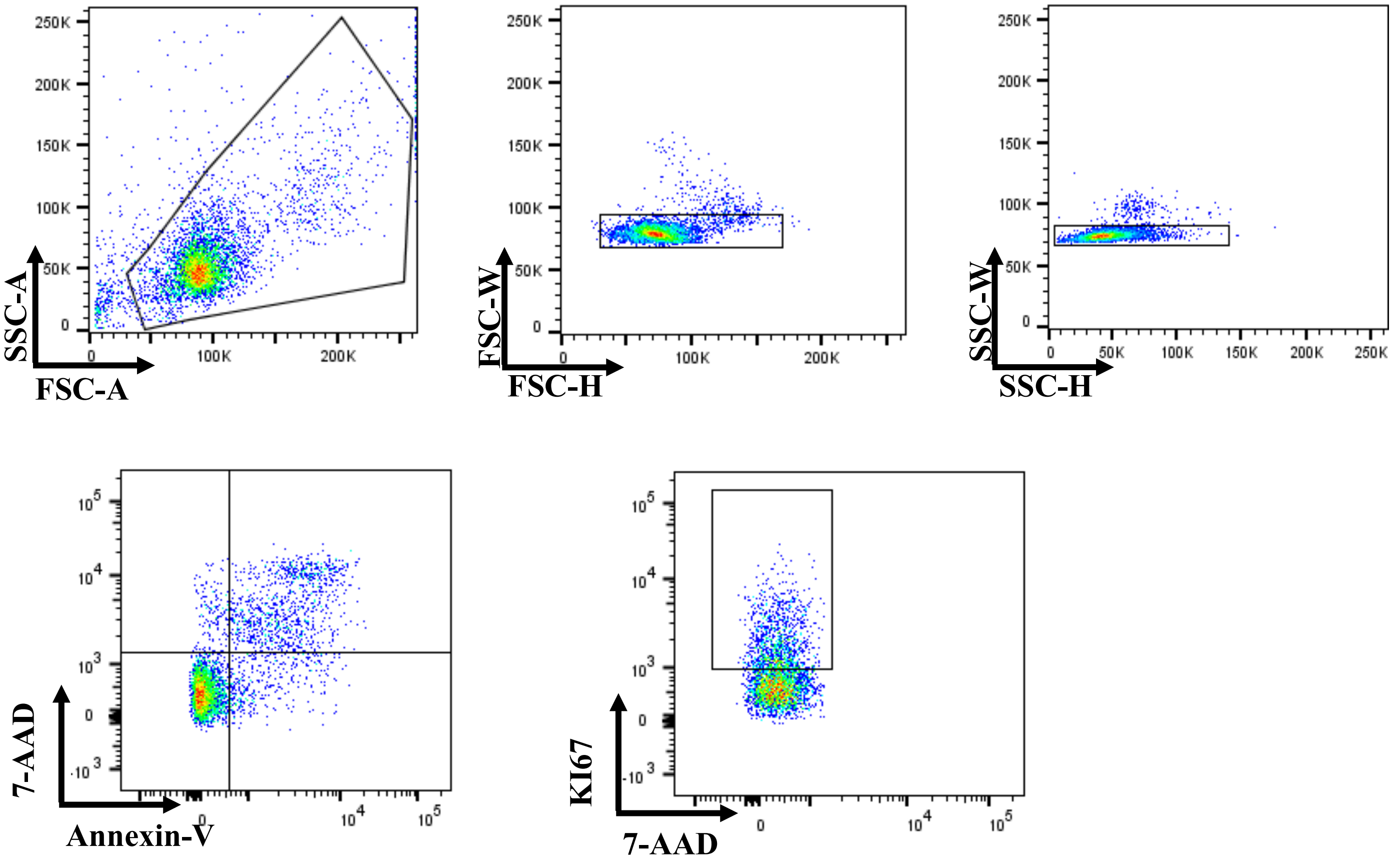

SSC-A
FSC-A
FSC-W
FSC-H
SSC-W
SSC-H
7-AAD
Annexin-V
KI67
7-AAD

d

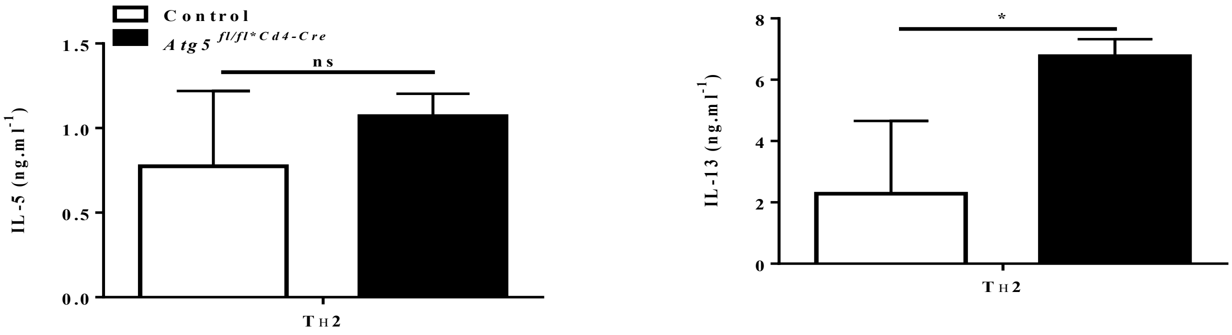

Control
Atg5 fl/fl*Cd4-Cre
ns
IL-5 (ng.ml-1)
TH2
*
IL-13 (ng.ml-1)
TH2

e

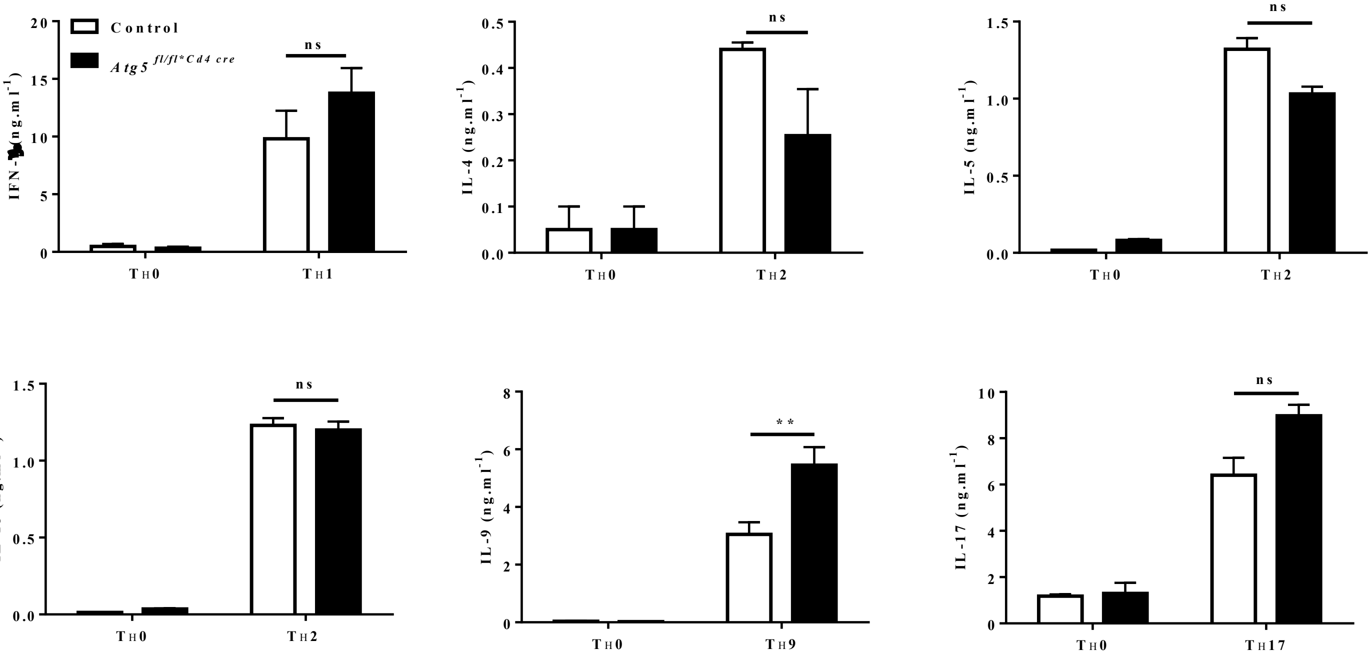

Control
Atg5 fl/fl*Cd4 cre
ns
**
IFN-γ (ng.ml-1)
IL-4 (ng.ml-1)
IL-5 (ng.ml-1)
IL-13 (ng.ml-1)
IL-9 (ng.ml-1)
IL-17 (ng.ml-1)
TH0
TH1
TH2
TH9
TH17

f

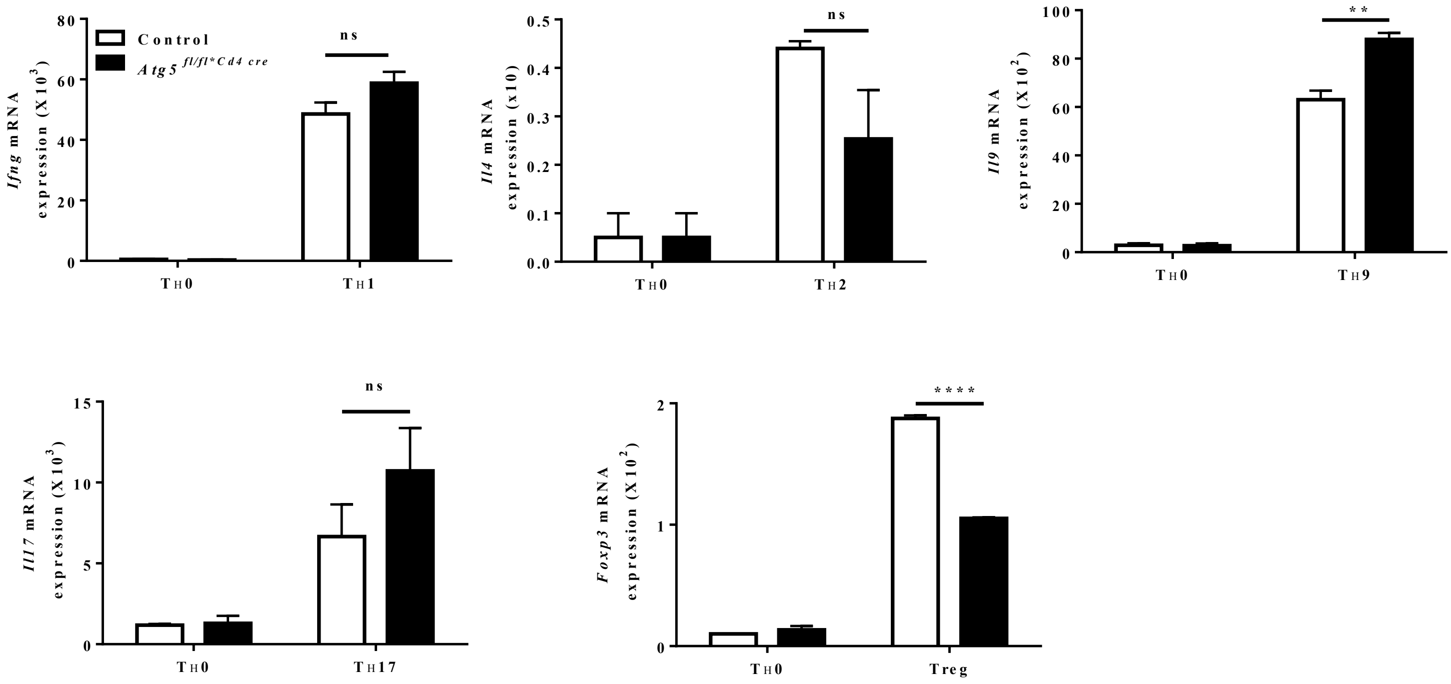

Control
Atg5 fl/fl*Cd4 cre
ns
**
****
Ifng mRNA expression (X10 3)
Il4 mRNA expression (x10)
Il9 mRNA expression (X10 2)
Il17 mRNA expression (X10 3)
Foxp3 mRNA expression (X10 2)
TH0
TH1
TH2
TH9
TH17
Treg

g

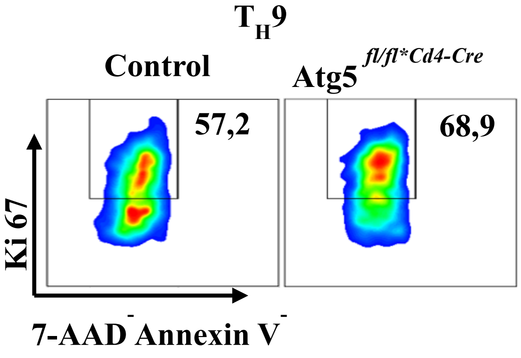

TH9
Control
Atg5 fl/fl*Cd4-Cre
57,2
68,9
Ki 67
7-AAD- Annexin V-

h

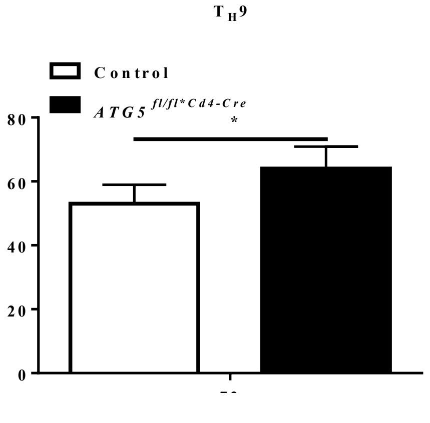

TH9
Control
ATG5 fl/fl*Cd4-Cre
*
Ki 67%

i

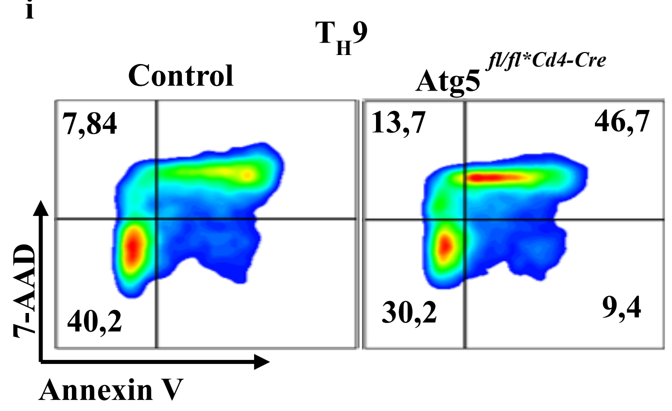
TH9
Control
Atg5 fl/fl*Cd4-Cre
7,84
40,2
13,7
46,7
30,2
9,4
7-AAD
Annexin V

j

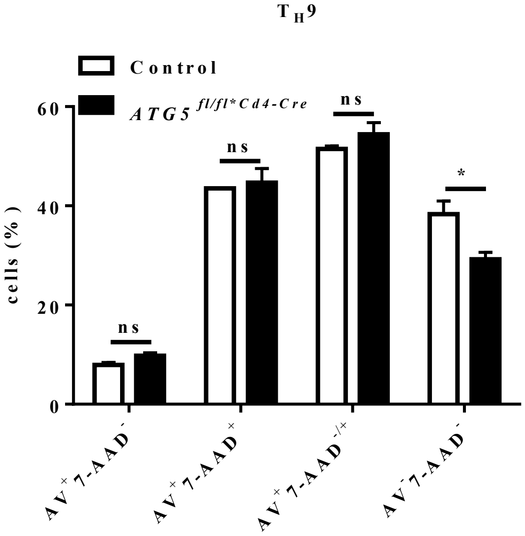
TH9
Control
ATG5 fl/fl*Cd4-Cre
cells (%)
60
40
20
0
ns
ns
ns
*
AV+ 7-AAD-
AV+ 7-AAD+
AV+ 7-AAD-/+
AV- 7-AAD-

k

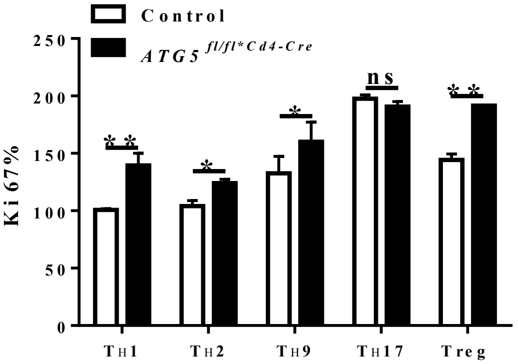
Control
ATG5 fl/fl*Cd4-Cre
Ki 67%
250
200
150
100
50
0
**
*
*
ns
**
TH1
TH2
TH9
TH17
Treg

l

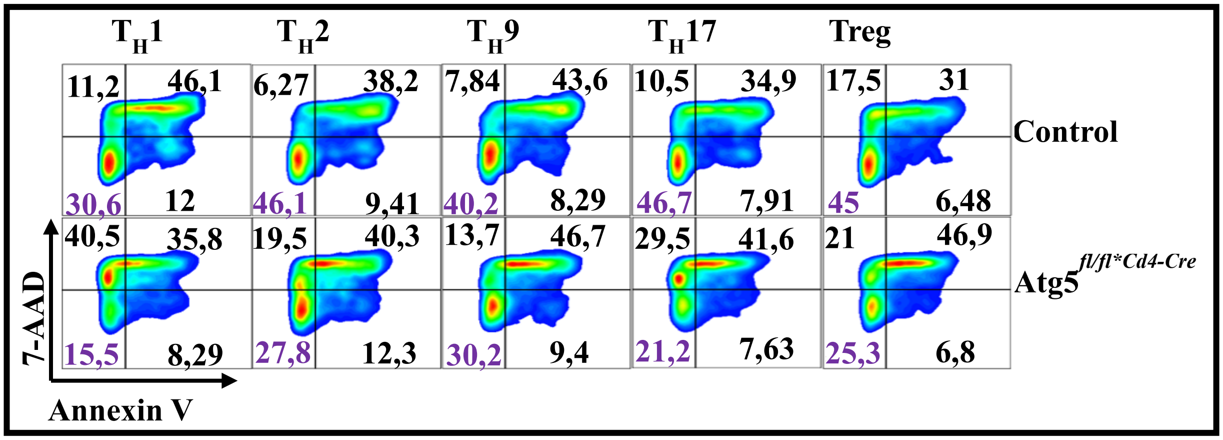
TH1
TH2
TH9
TH17
Treg
11,2
46,1
30,6
12
6,27
38,2
46,1
9,41
7,84
43,6
40,2
8,29
10,5
34,9
46,7
7,91
17,5
31
45
6,48
Control
40,5
35,8
15,5
8,29
19,5
40,3
27,8
12,3
13,7
46,7
30,2
9,4
29,5
41,6
21,2
7,63
21
46,9
25,3
6,8
Atg5 fl/fl*Cd4-Cre
7-AAD
Annexin V

m

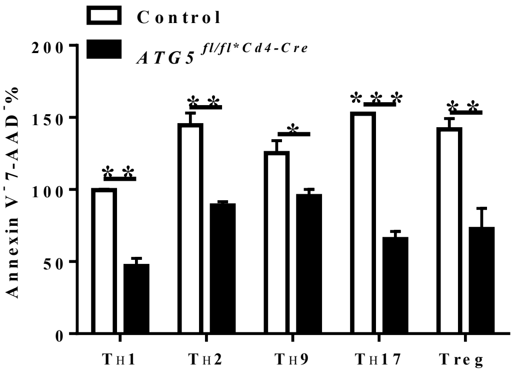

Control
ATG5 fl/fl*Cd4-Cre
Annexin V⁻ 7-AAD⁻ %
200
150
100
50
0
**
**
*
***
**
TH1
TH2
TH9
TH17
Treg

n

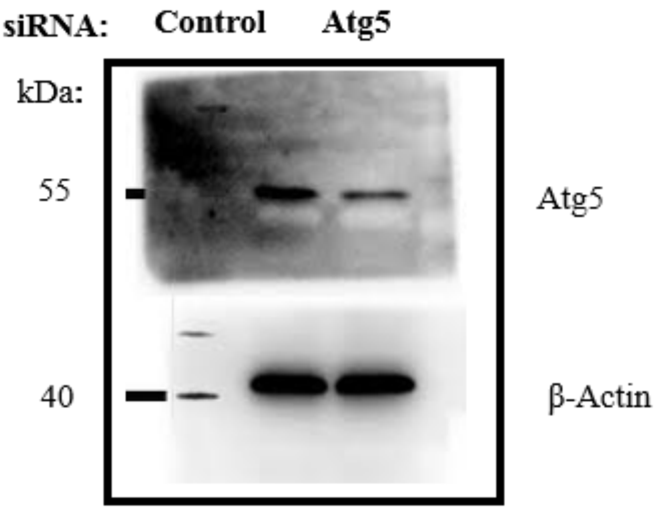

siRNA:
Control
Atg5
kDa:
55
40
Atg5
β-Actin

o

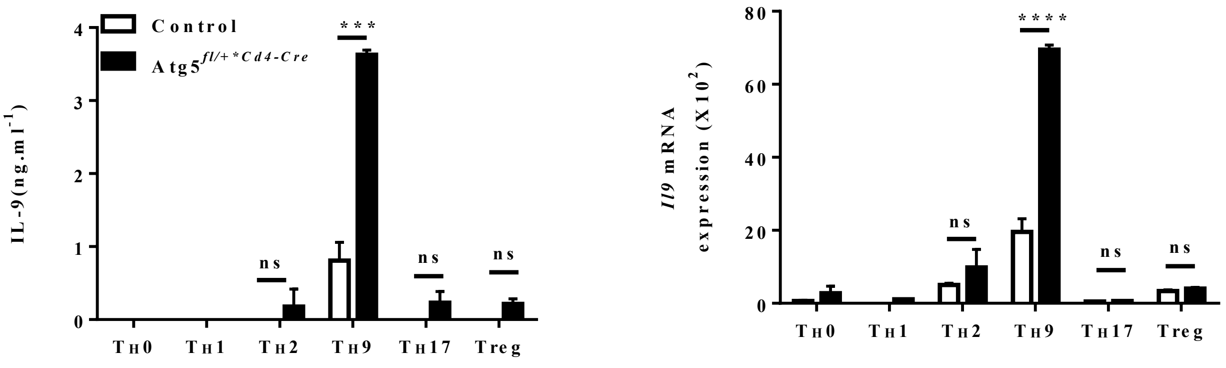

Control
Atg5 fl/+*Cd4-Cre
IL-9(ng.ml⁻¹)
4
3
2
1
0
ns
***
ns
ns
TH0
TH1
TH2
TH9
TH17
Treg
Il9 mRNA expression (X10²)
80
60
40
20
0
****
ns
ns
ns
TH0
TH1
TH2
TH9
TH17
Treg

Supplementary Figure 1: Atg5 selectively represses IL-9 expression.

Gating strategies for FACS analyses. a) Cell-sorting strategy for naïve $CD4^+CD62L^{hi}$ T cells. b) Gating strategies for analysis of T_H1, T_H2, T_H9 and T_H17 cells (single cells, live, $CD4^+$) in **Fig. 1c,d**; **Fig. 3d** and **Fig. 5l**. c) Gating strategies to study apoptosis and proliferation in T_H1, T_H2, T_H9, T_H17 and Treg cells with 7-AAD, annexin-V and Ki67 stainings shown in **Supplementary Fig. 1f, h, k** and **Supplementary Fig. 3a, c.** d) Cell-sorted naïve $CD4^+CD62L^{hi}$ $CD44^{lo}$ T cells strategy were isolated from $Atg5^{fl/+*CD4\text{-}Cre}$ and $Atg5^{fl/fl*CD4\text{-}Cre}$ mice and differentiated into T_H2 cells for 72 hours, IL-5 and IL-13 expression was assessed by ELISA. Experiment performed twice. (Mean +s.d.) NS, not significant; P>0.05; *P<0.05; unpaired Student's t-test. e and f) Cell-sorted naïve $CD4^+CD62L^{hi}$ $CD44^{lo}$ T cells were isolated from control $Atg5^{fl/fl}$ mice transduced with a Cre overexpressing vector or a control vector and differentiated into T_H0, T_H1, T_H2, T_H9, T_H17, and Treg cells in the presence of plate-bound anti-CD3 and anti-CD28 antibodies. Top panel: ELISA of IFN-γ, IL-4, IL-5, IL-13, IL-9 and IL-17 in supernatants of T_H0, T_H1, T_H2, T_H9, and T_H17 cells differentiated for 72 hours. Bottom panel: qRT-PCR analysis of *Ifng*, *Il4*, *Il9, Il17* and *Foxp3* mRNA in T_H0, T_H1, T_H2, T_H9, T_H17, and Treg cells differentiated for 48 hours; results were normalized to the expression of *Actb* and are presented relative to control T_H0 cells. Experiment performed twice. Mean (+sd.), NS, not significant; P>0.05; *P<0.05; **P<0.01;*** P<0.001 two-way ANOVA test. Cell-sorted naïve $CD4^+CD62L^{hi}$ $CD44^{lo}$ T cells were isolated from WT as control and $Atg5^{fl/fl*CD4\text{-}Cre}$ mice and differentiated into T_H9 cells for 72 hours. g) Cells were stained with Ki67 and analysed by flow cytometry. h) Graphic representation of flow cytometry analysis. Experiment performed twice. Mean (+sd.), *P=0.037 paired T-test two-tailed. i) Cells were stained with annexin V and 7-AAD and analysed by flow cytometry. j) Graphic representation of flow cytometry analysis. Experiment performed twice. Mean (+sd.), NS, not significant; P>0.05; *P<0.05; two-way ANOVA test. Cell-sorted naïve $CD4^+CD62L^{hi}$ $CD44^{lo}$ T cells were isolated from WT and conditional Atg5-deficient ($Atg5^{fl/fl*CD4\text{-}Cre}$) mice and differentiated into T_H1, T_H2, T_H9, T_H17, and Treg cells in the presence of plate-bound anti-CD3 and anti-CD28 antibodies (2 $\mu g.ml^{-1}$) for 72 hours. k) Cells were stained with Ki67 and analysed by flow cytometry at 72 hours of differentiation. The percentage of $Ki67^+$ WT cells in the T_H1 condition was set as 100%. The ratio of proliferating $CD4^+$T cells cultured in other conditions versus the WT T_H1 condition was calculated and shown. Experiment performed twice. l) Cells were stained with annexin V and 7–aminoactinomycin D (7-AAD) and analysed by flow cytometry. m) Flow cytometry results are shown in a graph where live cells were defined as annexin V^- and $7\text{-}AAD^-$ and the percentage of live WT cells in the T_H1 condition was set as 100%. The ratio of surviving $CD4^+$T cells cultured in other conditions versus the WT T_H1 condition was calculated and shown. Experiment performed twice. Mean (+sd.), NS, not significant; P>0.05; *P<0.05; **P<0.01;*** P<0.001 two-way ANOVA test. n) Full WB of T_H9 cells transfected with control siRNA or Atg5 siRNA with the marker position corresponding to **Fig. 1d** o) Cell-sorted naïve $CD4^+CD62L^{hi}$ $CD44^{lo}$ T cells were isolated from $Atg5^{fl/+*CD4\text{-}Cre}$ and $Atg5^{fl/fl*CD4\text{-}Cre}$ mice and differentiated T_H0, T_H1, T_H2, T_H9, T_H17, and Treg cells in the presence of plate-bound anti-CD3 and anti-CD28 antibodies. Cells were analysed by ELISA of IL-9 in supernatants of T_H0, T_H1, T_H2, T_H9, T_H17, and Treg cells differentiated for 72 hours and qRT-PCR analysis of *Il9* mRNA in T_H0, T_H1, T_H2, T_H9, T_H17, and Treg cells differentiated for 48 hours; results were normalized to the expression of *Actb* and are presented relative to control T_H0 cells. Shown is a typical experiment out of two. Mean (+sd.), NS, not significant; P>0.05; *P<0.05; two-way ANOVA test.

Supplementary Fig.2

a

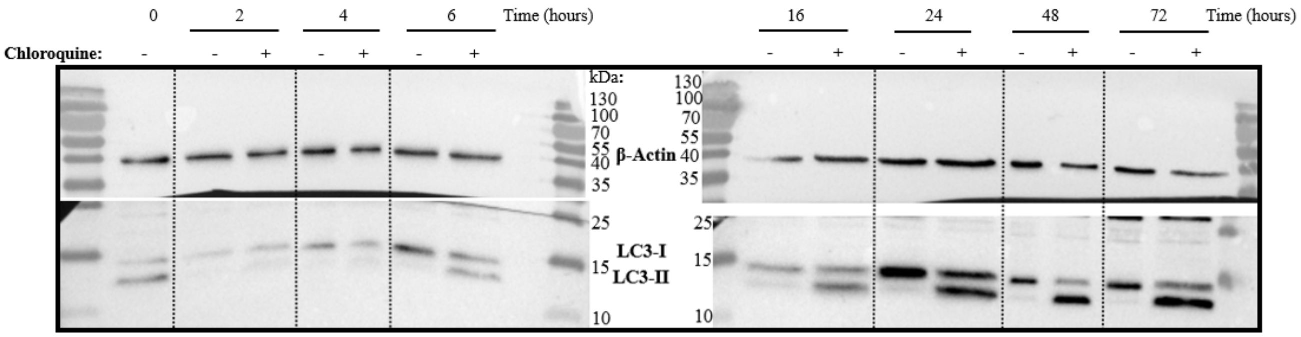

b

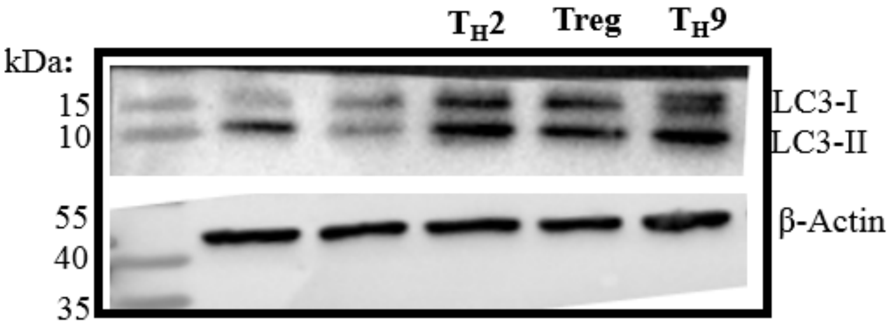

c

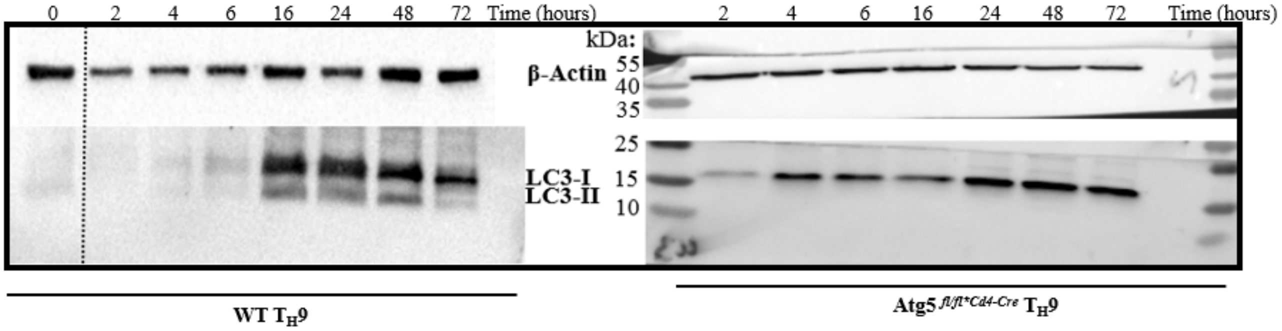

Supplementary Figure 2: Autophagy is active during T_H9 cell differentiation

a) Full immunoblot showing the conversion from endogenous LC3-I to LC3-II, the autophagosome marker, during T_H9 cell differentiation in the presence and in the absence of chloroquine corresponding to **Fig. 2a**. b) Full immunoblots of LC3-II in T_H2, Treg and T_H9 cells corresponding to **Fig. 2c**. c) Full immunoblots of LC3-II in Atg5 deficient cells compared to WT cells during T_H9 cell differentiation corresponding to **Fig. 2d**.

Supplementary Fig. 3

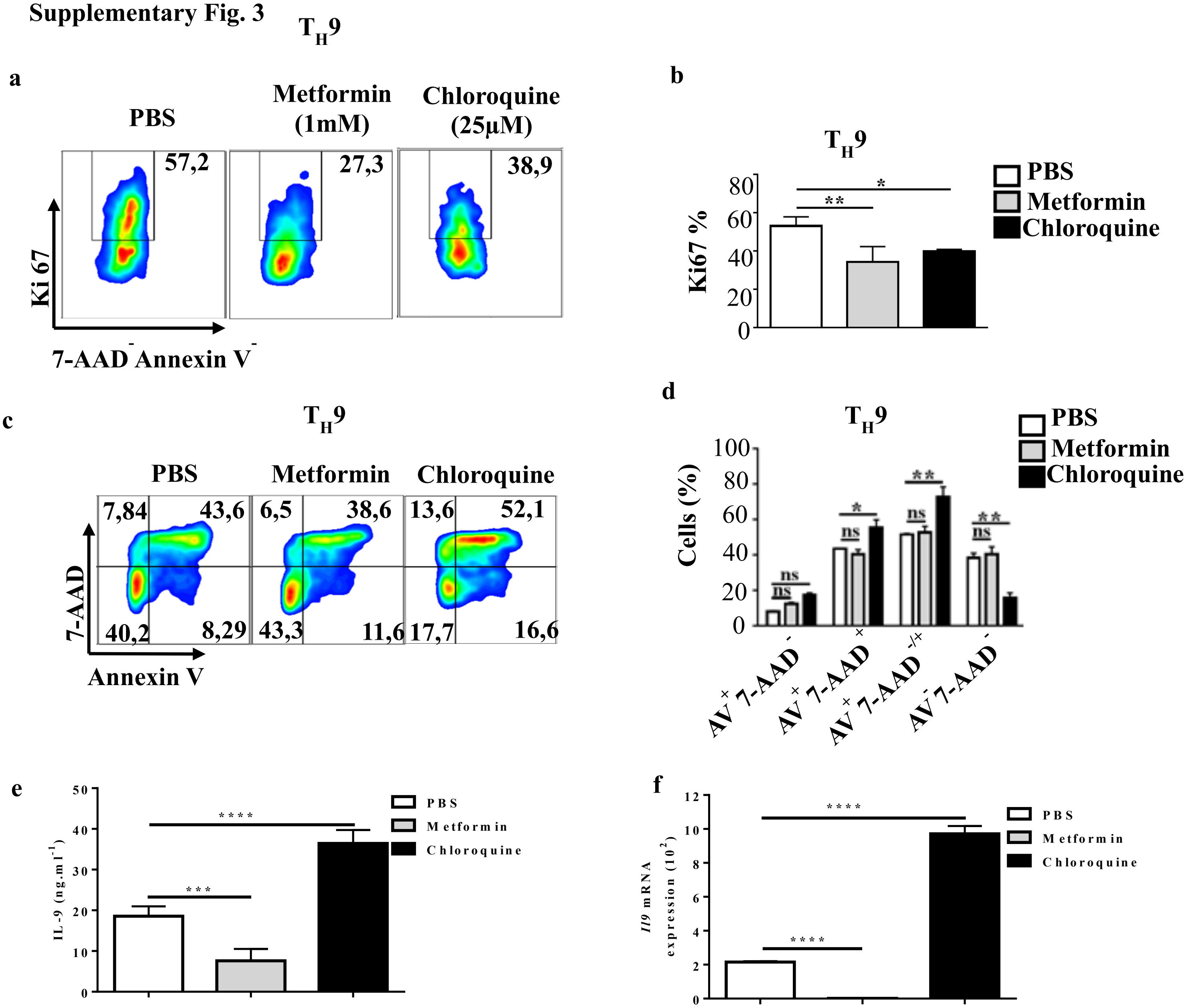

Supplementary Figure 3: Pharmacological modulation of autophagy affects T_H9 cell proliferation and survival

Cell-sorted naïve $CD4^+CD62L^{hi}$ $CD44^{lo}$ T cells were isolated from WT mice and differentiated into T_H9 cells for 72 hours in the presence or not of metformin at 1mM or chloroquine at 25µM. a and b) Cells were stained with Ki67 and analysed by flow cytometry. Experiment performed twice. Mean (+sd.), NS, not significant; P>0.05; *P<0.05; **P<0.01; One-way ANOVA test. c and d) Cells were stained with annexin V and 7-AAD and analysed by flow cytometry. Experiment performed twice. Mean (+sd.), NS, not significant; P>0.05; *P<0.05; **P<0.01; two-way ANOVA test. e and f) IL-9 expression was then analysed by ELISA and qRT-PCR, results were normalized to the expression of

Actb. Experiment performed twice. Mean (+sd.), NS, not significant; P>0.05; *P<0.05; **P<0.01; One-way ANOVA test.

Supplementary Fig. 4

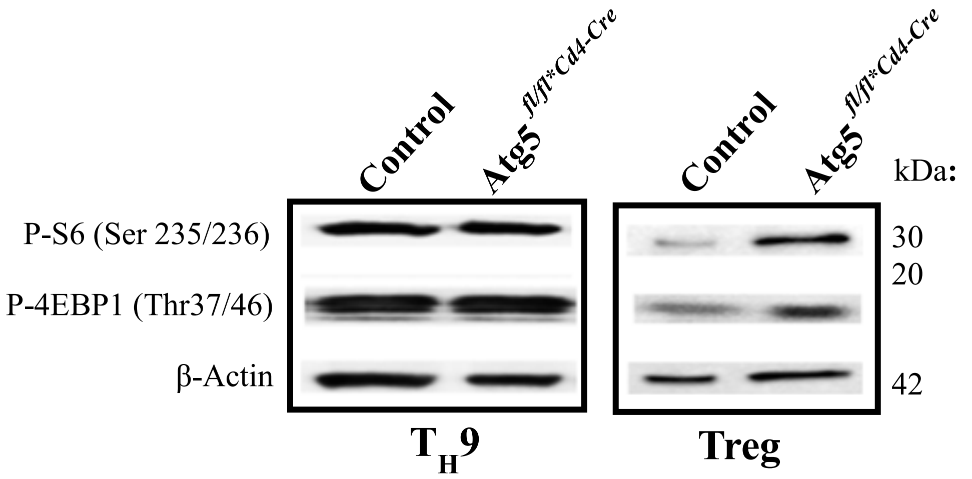

Supplementary Figure 4: Autophagy deficiency does not influence mTORC1 activity in T_H9 cells

Cell-sorted naïve $CD4^+CD62L^{hi}$ $CD44^{lo}$ T cells were isolated from control ($Atg5^{fl/+*CD4\text{-}Cre}$) and conditional *Atg5*-deficient ($Atg5^{fl/fl*CD4\text{-}Cre}$) mice and differentiated into T_H9 and Treg cells in the presence of plate-bound anti-CD3 and anti-CD28 antibodies for 72 hours. Immunoblot analysis of p-S6 and p-4EBP1 in *Atg5*-deficient T_H9 and Treg cells compared to control. Shown is a typical experiment out of two.

Supplementary Fig. 5

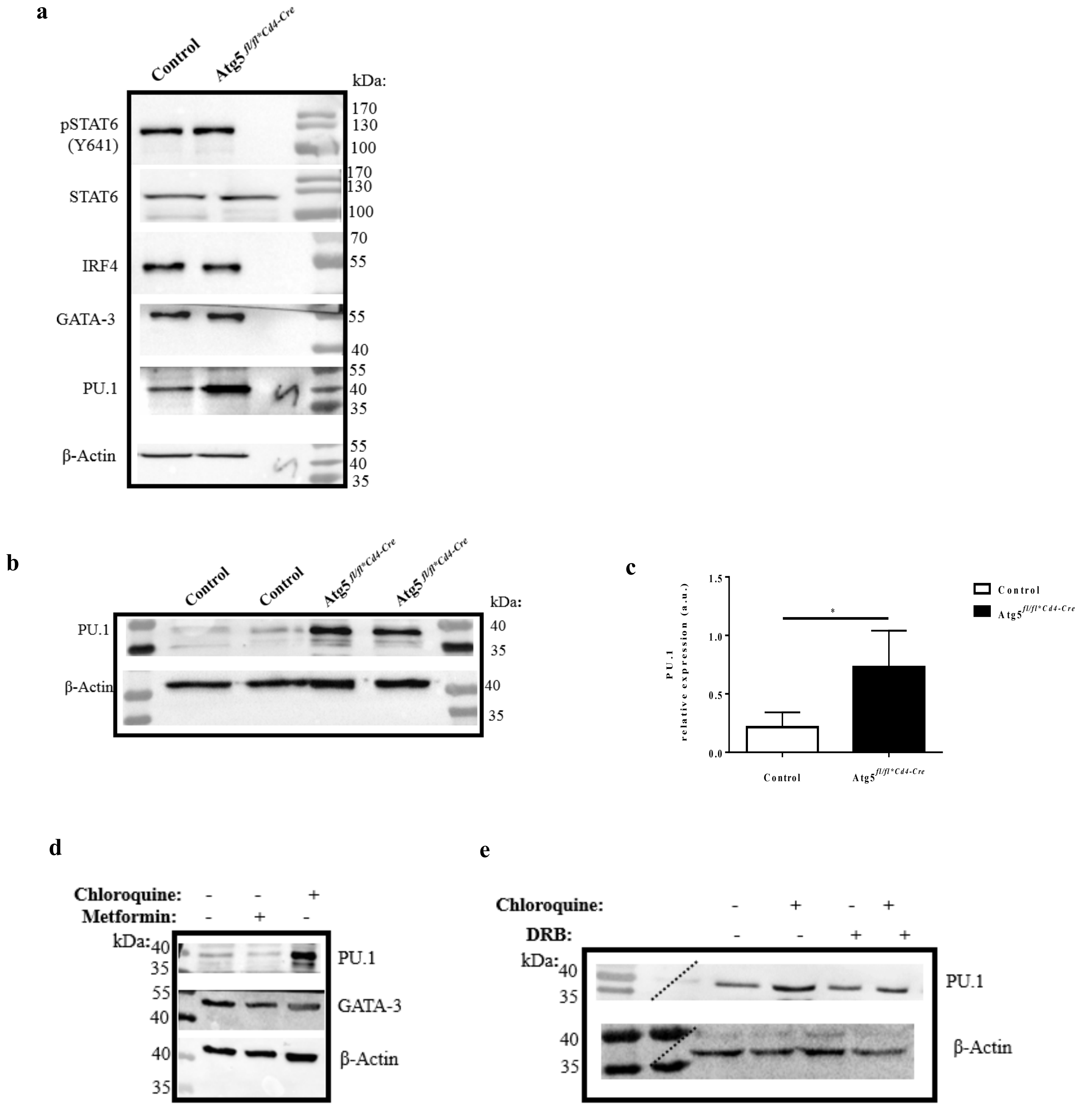

f

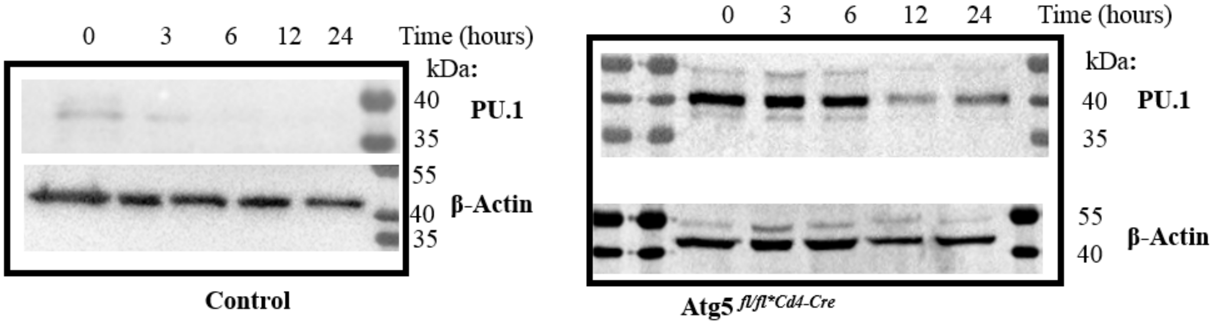

Supplementary Figure 5: Autophagy modulates PU.1 protein stability in T_H9 cells

a) Naïve $CD4^+$ T cells were isolated from $Atg5^{fl/+*CD4\text{-}Cre}$ and $Atg5^{fl/fl*CD4\text{-}Cre}$ mice and differentiated into T_H9 cells for 24h. Full Immunoblot of T_H9 transcription factors: pSTAT6, STAT6, IRF4, GATA-3 and PU.1 in *Atg5*-deficent T_H9 cells compared to controls corresponding to **Fig. 4a**. b) Immunoblot of PU.1 in *Atg5*-deficent T_H9 cells compared to controls. Data is representative of three experiments. c) Quantification analysis of the western blot shown in a. Mean (+sd.), *P <0.05 unpaired Student's t-test. d) Naïve $CD4^+$ T cells were isolated and differentiated into T_H9 cells for 24h in the presence of metformin (1mM) or chloroquine (25µM). Full western blot of PU.1, GATA-3 and β-actin expression corresponding to **Fig. 4b**. e) Full western blot of PU.1 expression analysed at 8h of DRB treatment corresponding to **Fig. 4f**. f) Naïve $CD4^+$ T cells were isolated from $Atg5^{fl/+*CD4\text{-}Cre}$ and $Atg5^{fl/fl*CD4\text{-}Cre}$ mice and differentiated into T_H9 cells. Full immunoblots of PU.1 protein after being treated with $25µg.ml^{-1}$ cycloheximide to inhibit protein synthesis for 3, 6 and 12 hours corresponding to **Fig. 4g**.

Supplementary Fig. 6

a

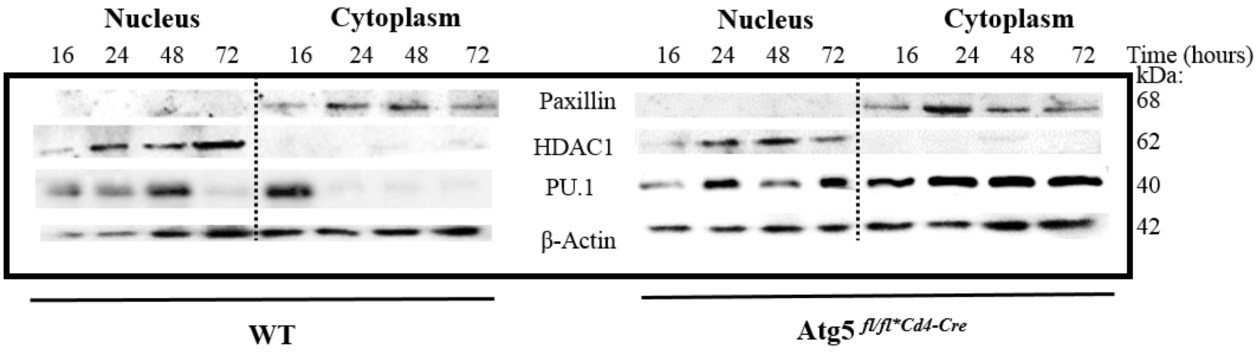

b

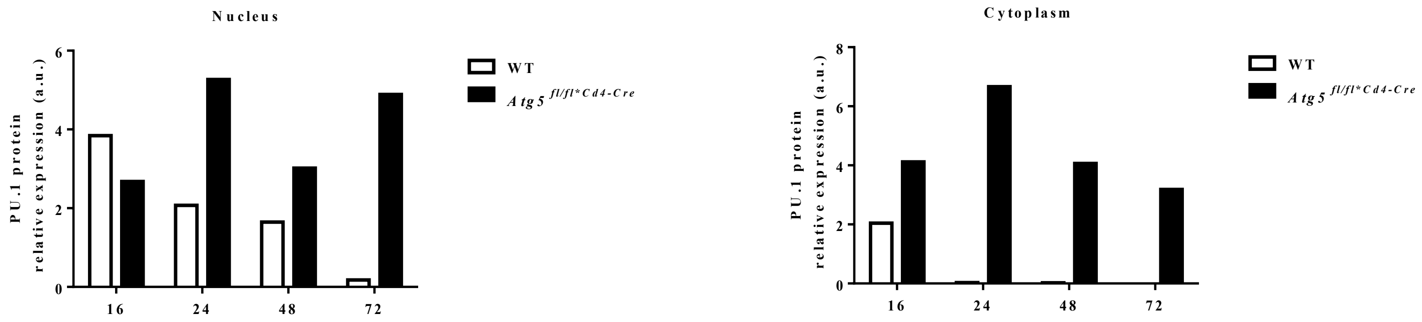

Supplementary Figure 6: ATG5 affects PU.1 expression during T_H9 cell differentiation

Naïve $CD4^+$ T cells were isolated from WT and Atg5$^{fl/fl*CD4\text{-}Cre}$ mice and differentiated into T_H9 cells. a) PU.1 protein expression and localization was assessed by western blot after subcellular fractionation at 8, 16, 48 and 72h of differentiation. b) Quantification analysis. Paxillin and HDAC1 were used as markers for the cytoplasmic and nuclear fraction respectively.

Supplementary Fig. 7

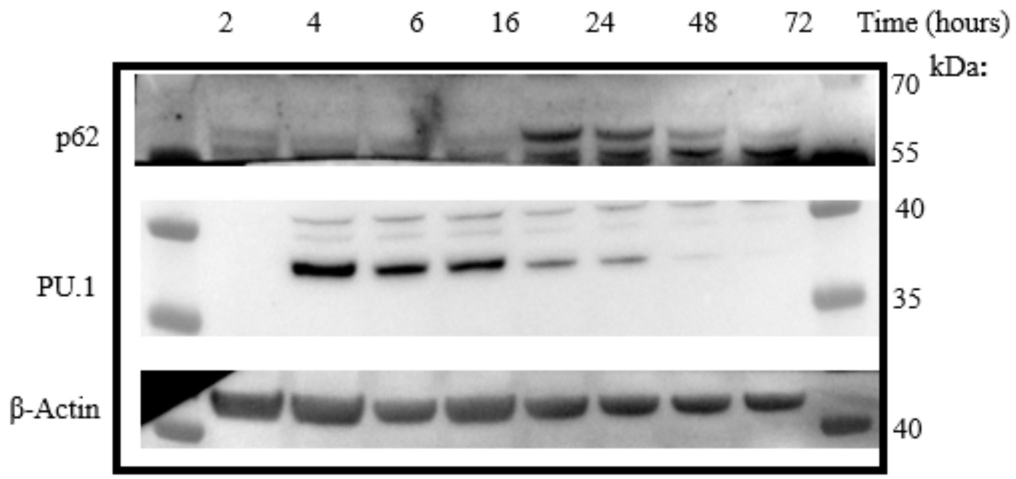

Supplementary Figure 7: Kinetic of p62 and PU.1 expression during T_H9 cell differentiation

Naïve $CD4^+$ T cells were isolated from WT mice and differentiated into T_H9 cells. Full western blot of the expression of p62 and PU.1 analysed at different time points during T_H9 cell differentiation corresponding to **Fig. 5a**.

Supplementary Fig. 8:

a

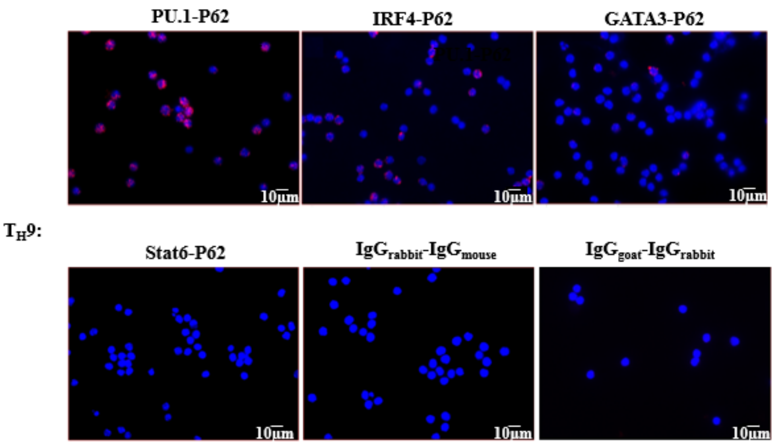

b

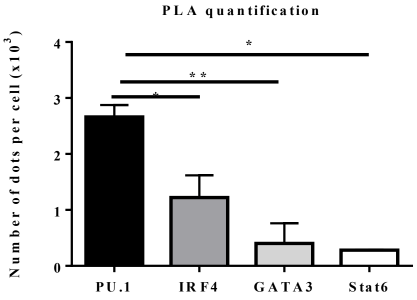

c

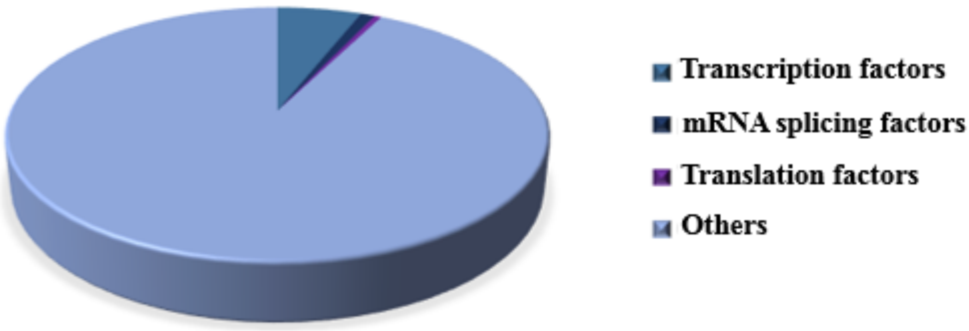

Supplementary Figure 8: Analysis of PU.1 complexes shows that p62 specifically interacts with PU.1 in T_H9 cells

a) PLA showing the interaction between p62 and T_H9 cell related transcription factors: PU.1, IRF4, GATA-3 and Stat6, as well as control antibodies. b) PLA quantification number of dots per cell out of 100 cells in two independent experiments. Mean (+sd.), NS, not significant; $P>0.05$; $*P<0.05$; $**P<0.01$; one-way ANOVA test. c) Mass spectrometry analysis of PU.1 complexes in T_H9 cells treated with chloroquine for 24 hours. The diagram shown was generated using the PANTHER software.

Supplementary Fig. 9

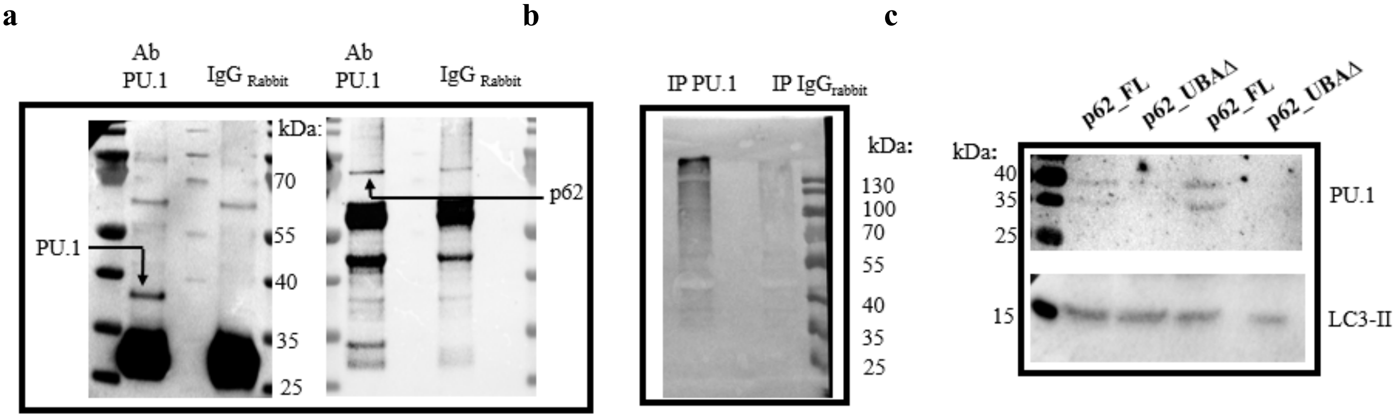

Supplementary Figure 9: Poly-ubiquitinated PU.1 protein is recruited by p62 in T_H9 cell.

a) Full immuno blot corresponding to co-immunoprecipitation experiment showing interaction of endogenous PU.1 and p62 in chloroquine-treated T_H9 cells (25µM), after 24h of differentiation corresponding to **Fig. 5f**. Shown is a typical experiment out of three.b) Full Immuno blot and marker corresponding to immunoprecipitation performed with anti-PU.1 antibody, followed by western blot detection using poly and mono-ubiquitination antibody to examine the presence of poly-ubiquitinated PU.1 in chloroquine-treated T_H9 cells (25µM), after 24h of differentiation corresponding to **Fig. 5g**. c) Full immune blot showing the binding of endogenous PU.1 and LC3-II by pull-down assay corresponding to **Fig. 5i**. Experiment performed twice.

Supplementary Figure 10:

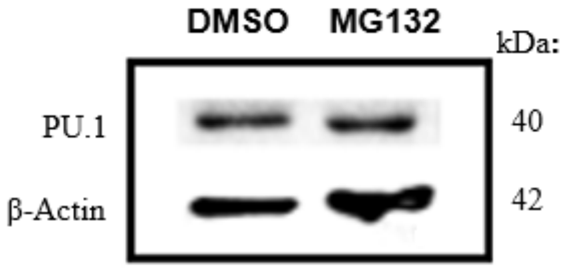

Supplementary Figure 10: The proteasome system does not influence PU.1 protein stability in T_H9 cells

T_H9 cells treated with DMSO or MG132 (2mM), a proteasome inhibitor for, 24h. PU.1 protein was assessed by western blot. Shown is a typical result out of two.

Supplementary Figure 11

a

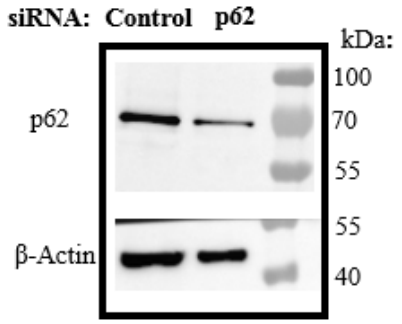

b

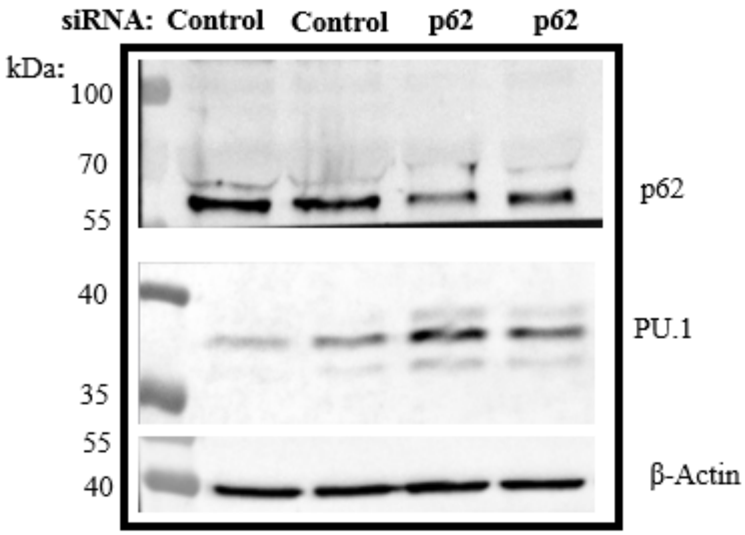

Supplementary Figure 11: p62 controls PU.1 protein stability in T_H9 cells

a) Full immune blots of p62 inhibition assessed by western blot 48 hour after transfection corresponding to **Fig. 5k**. b) Full immune blots of p62 inhibition and PU.1 protein expression corresponding to **Fig. 5m**.

Supplementary Figure 12

a

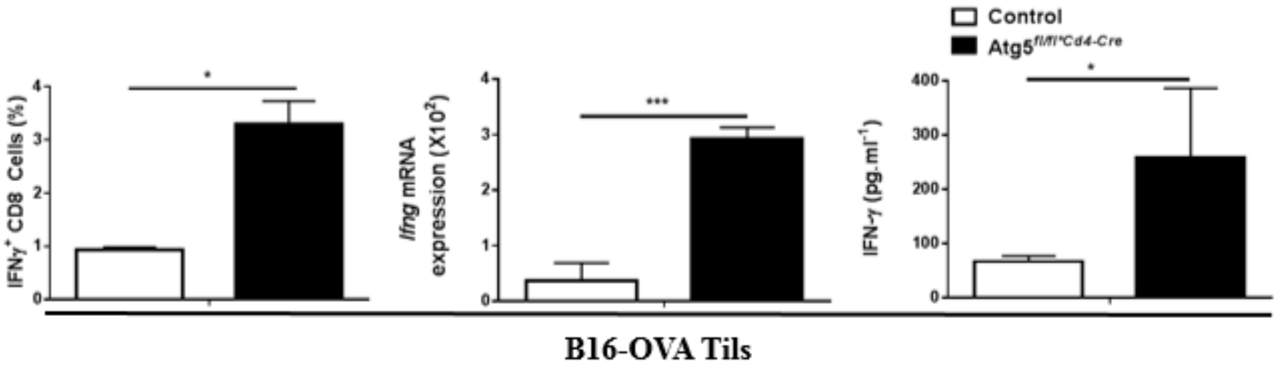

b

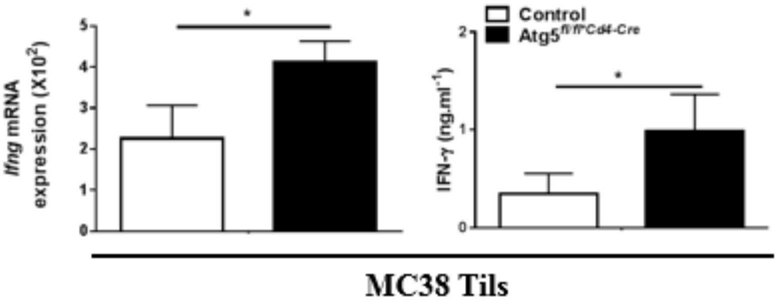

Supplementary Figure 12: *Atg5* deficiency in CD4 T cells specifically enhances TILs IL-9 expression in two different mouse tumor models *in vivo*

a) TILs of B16-OVA tumor bearing control and Atg5$^{fl/fl*CD4\text{-}Cre}$ mice isolated at day 20 after tumor cell injection and stimulated with the ovalbumin peptide $OVA_{257\text{-}264}$ for 24 hours. IFNγ expression from CD8 stimulated TILs was also analysed by qPCR, ELISA and FACS. b) MC38 TILs were stimulated with 50ng.ml^{-1} of PMA and 1µg.ml^{-1} of ionomycin for 24h. IFNγ expression was analysed by qPCR and ELISA (mean +sd., 5 mice per group, 2 independent experiments) Student's t-test.
